# Computer-aided design and 3-dimensional artificial/convolutional neural network for digital partial dental crown synthesis and validation

**DOI:** 10.1038/s41598-023-28442-1

**Published:** 2023-01-28

**Authors:** Taseef Hasan Farook, Saif Ahmed, Nafij Bin Jamayet, Farah Rashid, Aparna Barman, Preena Sidhu, Pravinkumar Patil, Awsaf Mahmood Lisan, Sumaya Zabin Eusufzai, James Dudley, Umer Daood

**Affiliations:** 1 Adelaide Dental School, The University of Adelaide, Adelaide, South Australia Australia; 2 Department of Electrical and Computer Engineering, North South University, Dhaka, Bangladesh; 3 Restorative Dentistry Division, School of Dentistry, International Medical University Kuala Lumpur, 126, Jalan Jalil Perkasa 19, Bukit Jalil, 57000 Bukit Jalil, Wilayah Persekutuan Kuala Lumpur, Malaysia; 4 School of Dental Sciences, Universiti Sains Malaysia, 16150 Kota Bharu, Kelantan Malaysia

**Keywords:** Computational biology and bioinformatics, Medical research, Engineering, Materials science

## Abstract

The current multiphase, invitro study developed and validated a 3-dimensional convolutional neural network (3D-CNN) to generate partial dental crowns (PDC) for use in restorative dentistry. The effectiveness of desktop laser and intraoral scanners in generating data for the purpose of 3D-CNN was first evaluated (phase 1). There were no significant differences in surface area [t-stat(df) = − 0.01 (10), mean difference = − 0.058, *P* > 0.99] and volume [t-stat(df) = 0.357(10)]. However, the intraoral scans were chosen for phase 2 as they produced a greater level of volumetric details (343.83 ± 43.52 mm^3^) compared to desktop laser scanning (322.70 ± 40.15 mm^3^). In phase 2, 120 tooth preparations were digitally synthesized from intraoral scans, and two clinicians designed the respective PDCs using computer-aided design (CAD) workflows on a personal computer setup. Statistical comparison by 3-factor ANOVA demonstrated significant differences in surface area (*P* < 0.001), volume (*P* < 0.001), and spatial overlap (*P* < 0.001), and therefore only the most accurate PDCs (n = 30) were picked to train the neural network (Phase 3). The current 3D-CNN produced a validation accuracy of 60%, validation loss of 0.68–0.87, sensitivity of 1.00, precision of 0.50–0.83, and serves as a proof-of-concept that 3D-CNN can predict and generate PDC prostheses in CAD for restorative dentistry.

## Introduction

The development of artificial intelligence (AI) took place in 1943, but the term “artificial intelligence” was coined at a session in Dartmouth in 1956^1^. Within this analogy, deep learning, neural networks, and machine learning are subsets of the AI. Machines can learn via building of algorithms solving predictive problems without human insights^2^. The neural networks (NN) used are mathematical non-linear models mimicking the human brain in traits of learning and decision making, stimulating human cognitive skills^3^. Such NNs can be complex with hidden layers can be trained to represent and predict multilayer perceptions processing data with deep learning^2^. Convolutional neural networks and artificial neural networks are the most used designs to process the data in planning prophylaxis, pivotal therapies, and projecting treatment costs^3^. Looking onto the near future, this technology will lead to the introduction of various new application areas within public domains in the form of smart assistants^4^. One of the areas that would benefit would be the field of dental medicine opening diverse opportunities of routine tasks that were initially performed by dental staff with improved quality in care^5,6^.

A priori, AI models have been commonly used for the mapping and finishing of tooth preparations and different prosthodontic applications. Computer-aided-design methods have also been used for tooth anatomy selection for automated dental restoration designs. Successful casting of metal frameworks, tooth shade selection and with porcelain shade matching have been recommended features of AI models^7^. Indirect restorations, partial dental crowns or PDCs (inlays and onlays) have recently begun generating popularity in the ‘minimally invasive dentistry’ movement. Reinforcing their proposed advantages, it has been established that gold and ceramic onlay preparations resulted in significantly less coronal tooth structure reduction compared with their full-coverage equivalents on the same tooth when performed by undergraduate students^8,9^. While commercially available digital solutions provided CAD assistance to dentists for digital inlay and onlay preparations, most free or open-source implementation were documented for dentures and larger prostheses as opposed to PDC^10,11^. Furthermore, the literature suggested that both the desktop laser scanners and intraoral scanners were accurate devices in their own rights and carried out specific features effectively^12–14^. The documentations did not however specify the ideal device to record input data for digitally recording tooth preparations for PDC and machine learning purposes. Considering the missing specific literature, previous reports of open-source CAD design were analyzed and modified to develop novel reconstruction workflows suitable for the current research. The workflows were revised and simplified, to eliminate the steep learning curve that are commonly reported by dentists welcoming clinical digitization^15–17^. Therefore, it was deemed appropriate that dentists designed the digital PDCs in CAD that would then be used in the machine learning process.

There are various impediments associated with AI or any other technology introduced. Recent medical and dental development of technologies have been expensive and were accompanied by unspecified patient compliance and acceptance amongst dental professionals^18,19^. Machine learning and neural networks have been proven to be successful in classifying and grouping data in a multitude of different fields of medicine and dentistry. In the past there have been a few documented methods of tooth preparation detection in teeth from radiographic image analysis using deep learning^20^. However, the prospect of using 3-Dimensional (3D) data of partial dental tooth preparations until now has evaded translational possibilities with 3D medical machine learning still in its infancy^21^.

The current study aimed to generate an accurate, novel 3D dental prosthetic dataset for 3D convolutional neural network (3D-CNN) training purposes. To generate a novel prosthetic dataset, the first challenge was to obtain accurate scans of prepared teeth. Based on past experiments, this can be achieved by both a desktop laser scanning system and intraoral scanner with no significant difference in surface topography and precision^12,13^. While both devices use imaging, infrared, and laser sensors to take thousands of images in a fraction of a second and compile them into 3D meshes, the key difference lies in the level of triangulation or magnification, with intraoral scanners providing larger magnification^12,13^. However, no study evaluated the system’s accuracy on partial dental crowns and therefore in phase 1, the two systems were virtually compared for which of the two generated more accurate 3D models relevant for the current prosthetic design.

The second challenge lay in that dental prostheses can be designed using both free and commercial software, with the most prominent differences being the number of features relevant to dentistry and ease of use for the human operator. As no previous study documented a comparison between the two options for partial dental crowns, in phase 2, the current study recruited two dentists with no previous computer aided design (CAD) experience and trained them on the workflows to design partial dental crowns on both the free and commercial CAD system. The scanned 3D models were augmented according to published medical AI practices^22^ and super-sampled to produce a larger dataset.

Therefore, the aim of the current study was to analyze different digital workflows to identify the most accessible workflow that can generate accurate novel digital data of partial dental crowns for 3D machine learning purposes. It was hypothesized that digital workflows could generate accurate dental data of partial dental crowns for 3D machine learning purposes using the proposed workflows.

## Results

The development of the convolutional neural network was possible using 3D STL models of tooth preparations of partial dental crowns.

### Phase 1

All assumptions for normality were met and an independent sample t-test was carried out. There were no significant differences in surface area [t-stat(df) = − 0.01 (10), mean difference = − 0.058, *P* > 0.99] and volume [t-stat(df) = 0.357(10), mean difference = 21.25, *P* = 0.375]. HD values ranged between − 0.02 to 0.10 mm with DSC ranging between 0.90 to 0.98. Intraoral scans produced greater volumetric details (343.83 ± 43.52 mm3) in comparison to desktop laser scanning (322.70 ± 40.15 mm3).

### Phase 2

A 3-factor ANOVA produced significant differences for MSA (F-stat = 111.28, *P* < 0.001) (Table 1) and VV (F-stat = 112.91, *P* < 0.001) (Supplementary Table 1) when type of prosthesis was an independent factor with no significant differences in the other factors. There were no significant interaction effects among the 3 independent factors. However, workflow 1 generated substantially larger surface area (129.63–140.30 mm^2^) and volumes (38.20–39.16 mm^3^) for inlays for both operators. Analyses of HD (Supplementary Table 2) and DSC (Supplementary Table 3) demonstrated significant interchangeable interactions between virtual workflow, clinical operator and type of prosthesis being designed. Workflow 2 produced greater DSC (0.85–0.95) for onlays. One 3D model was digitally corrupted at the time of spatial overlap analysis, and therefore the mesh and its three counterparts generated by the clinicians from the other workflows were removed prior to statistical evaluation to maintain the integrity of the report.

**Table 1:** Analysis of variance (n = 116) for mesh surface area.

List of independent factors:		
**1. Type of Prosthesis**: F(df) = 111.279(1), *P* < 0.001 Inlay = Mean ± SD = 112.44 ± 46.05 Onlay = Mean ± SD = 291.65 ± 117.62 **2. Virtual Workflow**: F(df) = 1.355(1), *P* = 0.247 Workflow 1 (3matics) = Mean ± SD = 214.59 ± 120.21 Workflow 2 (Meshmixer) = Mean ± SD = 195.68 ± 134.36 **3. Clinical Operator**: F(df) = 0.018(1), *P* = 0.894 Operator 1 = Mean ± SD = 206.21 ± 127.48 Operator 2 = Mean ± SD = 204.07 ± 128.19		

	Operator 1 *(Mean* ± *SD)*	Operator 2 *(Mean* ± *SD)*
Inlay		
Workflow 1	140.30 ± 60.739	129.63 ± 40.131
Workflow 2	90.298 ± 26.026	89.550 ± 27.355
Onlay		
Workflow 1	285.95 ± 122.64	291.87 ± 119.51
Workflow 2	296.17 ± 117.45	292.62 ± 122.96

Interaction effect		
1. Type of Prosthesis vs. Virtual Workflow: F(df) = 2.211(1), *P* = 0.140 2. Type of Prosthesis vs. Clinical Operator: F(df) = 0.041(1), *P* = 0.840 3. Virtual Workflow vs. Clinical Operator: F(df) < 0.001(1), *P* = 0.995 4. Type of Prosthesis vs Virtual Workflow vs Clinical Operator: F(df) = .081(1), *P* = 0.776		

### Phase 3

The 30-specimen dataset produced a maximum validation accuracy of 60% in determining the type of prosthesis required for each tooth preparation. Validation loss of 0.8748 was seen in tooth preparation dataset and 0.6832 in prosthetic dataset. (Supplementary Fig. 1) Sensitivity was 1.00 for both datasets with 3D tooth preparation dataset producing a precision of 0.50 while 3D prostheses producing 0.83. Apart from precision, every accuracy indicator of the automatic segmentation revealed a distinction between the groups that was statistically significant (*P* < 0.05).

## Discussion

The current study aimed to investigate the most reliable workflows to generate novel 3D data for dental machine learning purposes and subsequently classify 3D models of partial dental crowns and their tooth preparations by training the model using multidisciplinary medical datasets. In addition, this was to validate an innovative AI-driven tool for time-efficient and precise automation. To the authors’ knowledge, this is the first study to have attempted the said approach. The AI-driven tool demonstrated high accuracy and a fast performance. While both scanning apparatuses in phase 1 are considered standard for their respective roles according to previous literature^20,21^, the current study found substantial differences between intraoral scans and desktop scans indicating that intraoral scans are more appropriate to detect the line angles and finish lines on the prepared teeth. The thin layer of titanium oxide applied may have affected the outcomes, but further validation is required to confirm this hypothesis. Unlike radiomic datasets, where clinical experience is critical and dictate the overall success of machine learning^23^, both workflows in phase 2 produced respectable results when operated by 2 dentists with little to no experience with dental CAD. however, surface reconstruction for inlays while Boolean subtraction for onlays generated more reliable outcomes supporting the age-old argument of commercial software being highly optimized for smaller details while open-source or crowd supported projects being more user accessible for general projects^9,10^.

Accuracy metrics are widely acknowledged for assessing AI segmentation quality. However, the time as a factor has received less attention throughout the process, which makes it a pivotal influencer when considering its clinical applicability and relevance. The current study was greatly limited by the size of the original dataset (n = 6) and the fitting of data in GPU memory optimization. 3D neural network applications require substantial graphical computing periods that is still being optimized for the inevitable era of augmented reality and virtual metaverse. To tackle this, transfer learning of similar data was investigated with robust techniques like application of 3D convolutional networks^24,25^. The lack of 3D data for machine learning in dental restorative sciences encouraged transfer learning from multidisciplinary lung datasets to train smaller dental dataset. The current work demonstrated that 3D images obtained from .stl file data can be directly fed into a 3D neural network model instead of regarding the 3D spatial information as a stacked input of 2D based methods^26^. A validation accuracy of 60% with a validation loss (the sum of errors made for each example in training or validation sets) below 1.00 on such a small dataset would indicate promising prospects for further development. Oversampling the data in CAD to increase sample size and appointing different practitioners to design the prostheses helped introduce minute variations on the 6 specimens which led to the production of 120 specimens. Future studies with increased dental sample size, with the current model transferred into the network, and application of the promising generative adversarial networks^21^ can potentially increase accuracy, lower issues of overfitting, while clinically allowing for more targeted machine learning applications in dental diagnostics and treatment planning.

The findings of the current report suggest that the 3D STL models of tooth preparations of partial dental crowns can be analyzed and processed using neural networks. However, the main error identified during the process for all groups tested was instances of under-estimation or under-evaluation. Such limitations may be explained by the presence of artifacts that can produce higher false-positive voxels or wide parameter adjustments commonly accompanying neural networks^27,28^. At the same time, these errors are unlikely to have detrimental impact in a clinical scenario and can be implemented in challenging and complex cases.

The conversion of solid objects into 3D data matrices creates the potential for segmentation, classification, and analysis of dental cavities, oral cysts, and neoplastic lesion from models generated directly from 3D radiographic imaging. Finally, accurate generation of fillings and crowns through autoencoders (i.e., neural networks that can compress and create meaningful information for decoding later) can be performed just by analyzing the cavity itself, completely mitigating the need for operator intervention in designing the prosthesis^29^. With the availability of a larger dataset and at a reduced graphics intensive workload, the use of autoencoders and 3D-CNNs can potentially negate the need for an expensive computer setup to run 3D machine learning application and make this technology commonplace.

## Conclusion

Within the limitations of the study, the findings of the current in-vitro study are as below:

1. Intraoral scans can produce more accurate surface texture details of teeth prepared for partial dental crowns than laser scanner-derived 3D images.
2. The study demonstrated that clinicians do not require expensive computer setups or experience with CAD to design virtual crown prostheses that are fit to facilitate machine learning.
3. The study serves as a proof of concept that both open-source and commercial CAD workflows can process virtual data for tooth preparations which are acceptable for machine learning in restorative dentistry.
4. 3D deep learning can generate and predict partial dental crown restorations appropriate for tooth preparations in dentistry.

## Materials and methods

This study was conducted in compliance with the World Medical Association Declaration of Helsinki on medical research. Informed consent was taken from all subjects providing tooth samples. All experimental protocols were approved by the Joint Ethical Committee/Institutional Review Board at International Medical University under Project No. 259/2020. The current experiment was designed and executed in 3 phases as summarized in Supplementary Fig. 2.

### Phase 1: scanning

Six extracted natural teeth were prepared for inlay and onlay restorations following standardized methodology. A mesio-occlusal distal cavity was prepared on the proximal surfaces. The bur was kept at 90 degrees to occlusal plane and slightly tilted laterally. The bur was in rotation when applied and did not stop rotation until and unless was removed from the tooth. The marginal ridge was thinned out before dropping into the proximal box. For the occlusal segment, the line and point angles were defined with buccal and lingual walls parallel to each other and at 90 degrees to the occlusal plane. The mesial and distal walls were divergent pulpo-occlusally. The bur was moved facially and lingually along the dentino-enamel junction and oriented according to the proximal wall directions. The gingival margins were extended gingivally^30^. The prepared teeth were coated in titanium dioxide spray to reduce surface reflection of ambient light for scanning^31,32^.

Each preparation was scanned once using an intra-oral scanner (3Shape; Trios) and once using a desktop laser scanner (3D Scanner Ultra HD; Next Engine Santa Monica). The scans were exported as standard tessellation language (STL) files and virtually quantitatively evaluated for likeness in surface contour, geometric and volumetric similarities by measuring four separate parameters^33,34^: mesh surface area (MSA), virtual volume (VV), Hausdorff’s distance (HD) and Dice Similarity co-efficient (DSC). MSA evaluated the surface contour, VV measured the volumetric similarities, HD broke the two objects into points and measured the number of interpoint mismatches while DSC measured the volumetric spatial overlap between the two objects. The scanning method that produced the best visual (Supplementary Fig. 3) and quantitative results was selected for phase 2.

### Phase 2: restoration design

**Figure 1:**
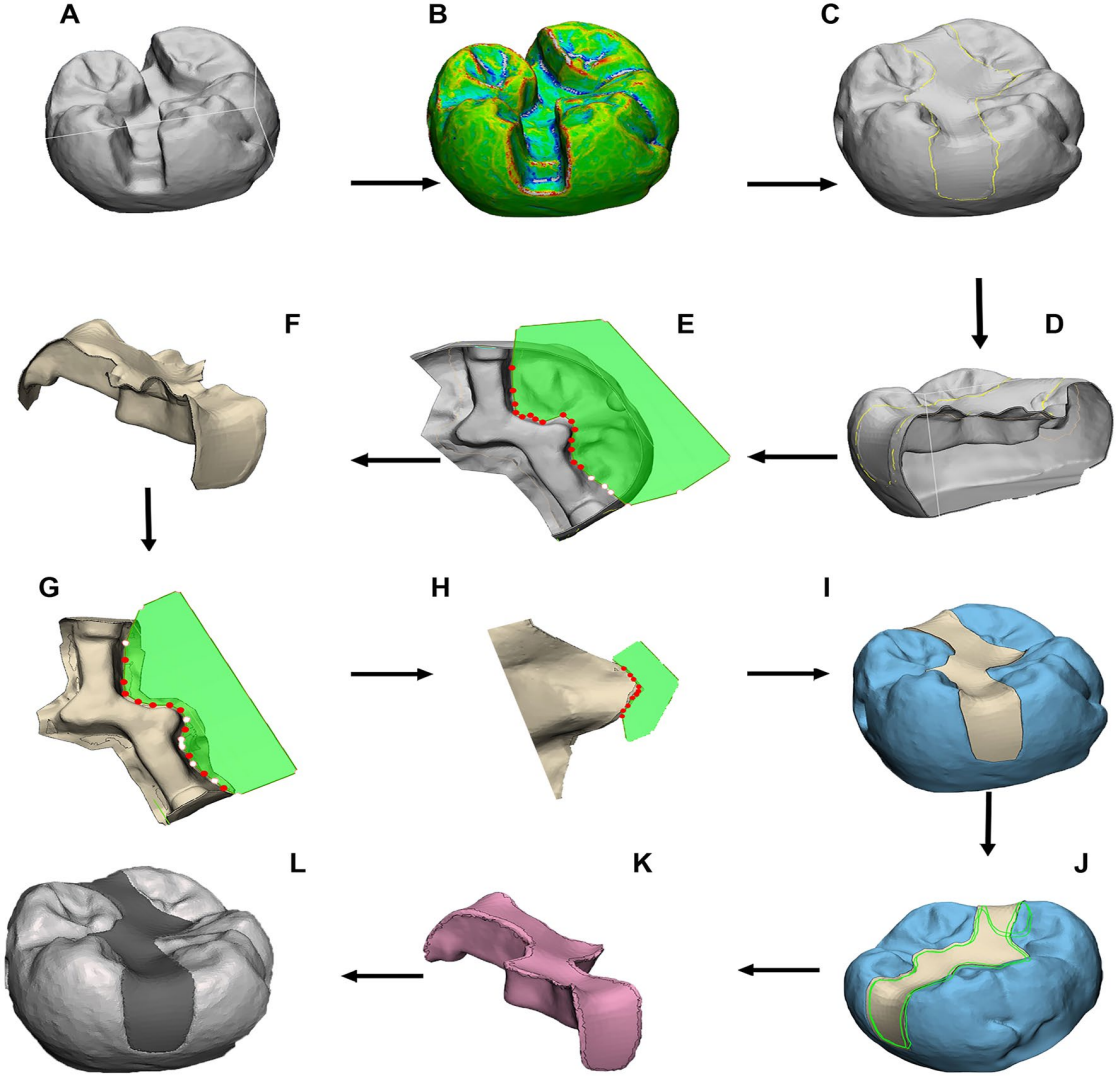
Workflow 1 for inlay design with respective commands: (**A**) 3D model loaded into 3-matics, (**B**) curvature analysis and curve creation on model surface, (**C**) surface reconstruction function to bridge defect , (**D**) hollow the model and trim down, (**E**) manual trimming of isolated segments, (**F**) apply wrap function, G) manually trim after wrapping, (**H**) manually trim any overhangs, (**I**) check fit onto 3D preparation model, (**J**) fine smoothing of edges, (**K**) final wrap and apply autofix function to fix any defects, (**L**) final output.

**Figure 2:**
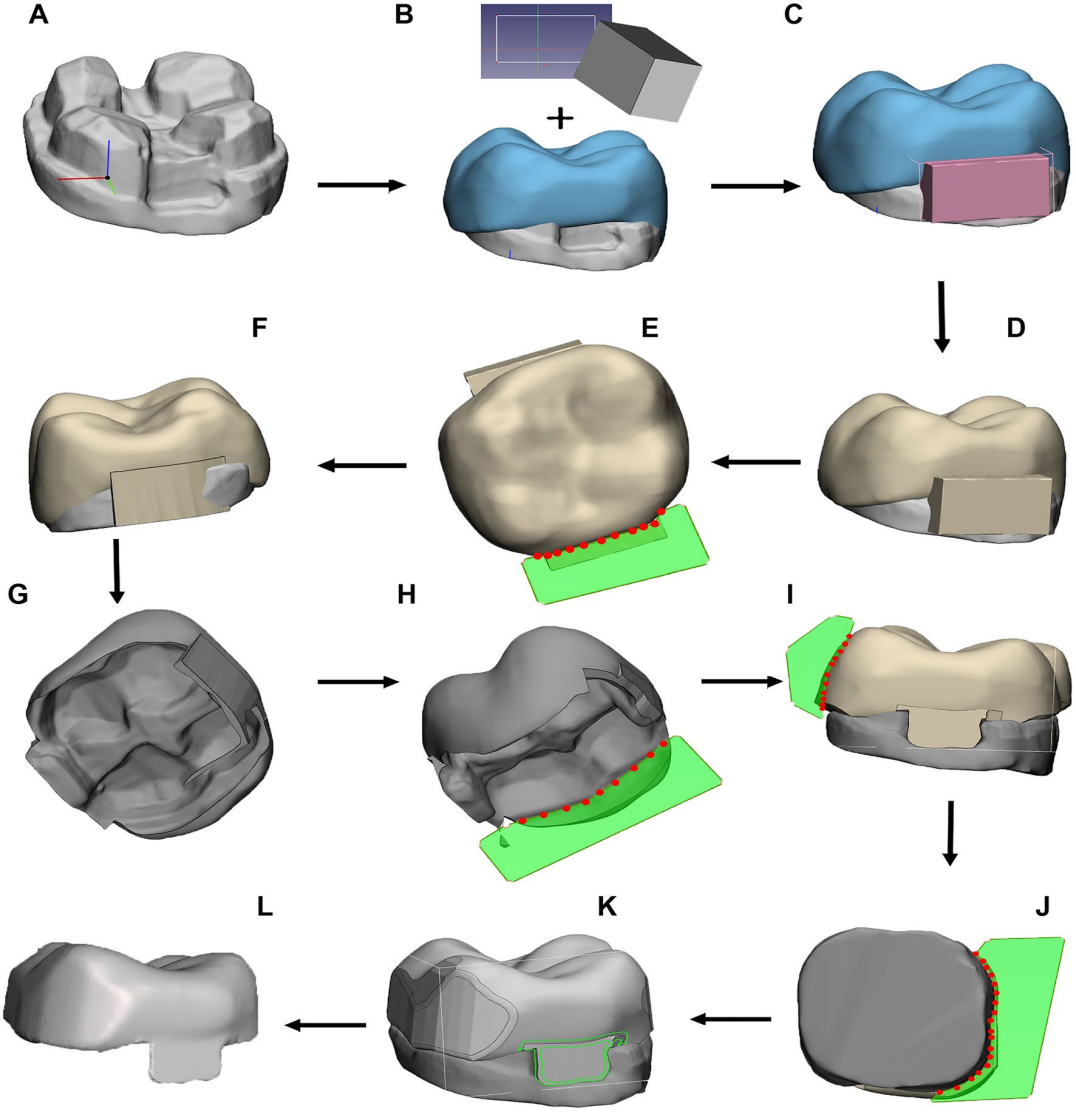
Workflow 1 for onlay design: (**A**) import 3D tooth preparation, (**B**) superimpose crown template, (**C**) import and scale block template, (**D**) perform Boolean union, (**E**) trim excess, (**F**) check contour for overhang, (**G**) perform Boolean intersection, (**H**) remove excess, (**I**) contour edges, (**J**) crown shaping through manual trim, (**K**) edge smoothing, (**L**) sculpt and smooth.

The tooth preparations were categorized according to the type of restoration (inlay or onlay) and two workflows were developed for each category of restoration: Workflow 1—Medical grade commercial software (3matics; Materialise NV); Workflow 2—Free software (Meshmixer; Autodesk Inc). Inlays for medical grade commercial software were designed following the ‘surface construction’ principles (Fig. 1), as documented in a previous literature^11,35^. The inlay design for free software (Supplementary Fig. 4) and onlay designs for both commercial (Fig. 2) and free software (Supplementary Fig. 5) followed the ‘Boolean subtraction’ principles, as previously documented^36^. Virtual crown templates were obtained from scanned physical restorations on dental casts and were used to reconstruct the onlay cusps^9,10,37^.

The process and commands for the restorations in both workflows were video recorded using low detail practice crown templates and documented in text (Supplementary file). The videos and template files were provided to two dentists with no prior experience in CAD-based rehabilitation to offset potential operator dependent biases^36^. The dentists practiced digital rehabilitation using the templates at their convenience for one week before being introduced to the actual crown templates as selected and designed by a certified prosthodontist. This process was followed to discourage memorized familiarity-induced fatigue^38^. The six specimens were digitally oversampled five times introducing incremental variations in tooth preparation size and depths to facilitate a machine learning model trained using a larger variation in tooth preparations. This resulted in 30 samples (Supplementary Fig. 6) assigned to each workflow, thereby producing 120 samples (60 from each dentist). This graphics intensive task of oversampling was undertaken on a computer (ROG Flow  X13; Asus) sporting an AMD Ryzen 7 5800HS processor, 16 GB of RAM, NVIDIA GTX 1650 max-Q design dedicated graphics and a thermal design power (TDP) of 30 W. However, to standardize the evaluation and keep it practically relevant, both practitioners designed all the prostheses on a personal computer (Idea pad Flex 5; Lenovo) with an intel Core i5 1135g7 processor, 8 GB of RAM and 512 GB of NVM.e solid state drive, no dedicated GPU, and a TDP of 15 W, reflecting an average modern day laptop computer of 2022. The workflows with the most consistent and favorable MSA, VV, HD and DSC values were evaluated, and the best performing workflows as determined by greater surface area, volumetric similarities, and spatial overlap, were selected to train the machine learning model in Phase 3.

### Phase 3: deep learning from 3D data

**Figure 3:**
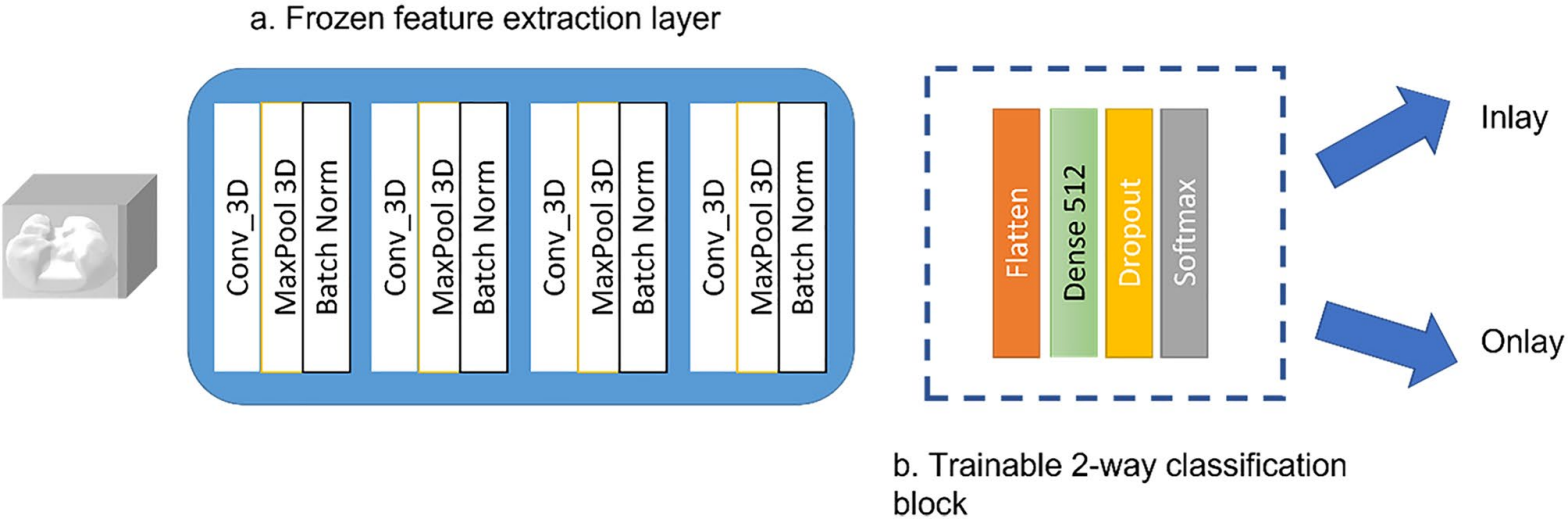
The 3D neural network.

30 specimens and their corresponding restorations from the best workflows were selected for transfer learning and machine learning application and sliced into 2D segments (Supplementary Fig. 7) and later appended into readable 3D matrix. A 17-layer 3D convolutional neural network (CNN) of four 3D layers implemented at a kernel size of 3 × 3 × 3^39^. The 17-layer CNN had a frozen feature extraction block consisting of 13 layers and a classification block consisting of four layers. The first two layers consisted of 64–128 and 256 filters with each CNN layer followed by a 2-stride max pool layer and a ReLU activation which ends with batch normalization (BN) layer. The classification block had 512 neurons of dense ‘Flatten’ layer followed by a 60% ‘Dropout’ layer and finally ‘SoftMax’ layer. The primary dataset for training was obtained from the Image CLEF Tuberculosis 2019 dataset^40^ and frozen after obtaining a 73.3% validation accuracy. 132,097 trainable parameters were transferred to train the current dataset. The dental dataset was broken down to 2:1 ratio for training and validation. Training was done for 100 epochs with planned termination set should validation accuracy not improve beyond 15 epochs. The developed 3D network has been highlighted in Fig. 3.

### Statistical analysis

Sample size estimation for a healthy evaluation for MSA, VV, HD, and DSC was carried out using G*Power^41^. A large effect size f = 0.40, α = 0.05, power = 0.85 determined a minimum sample size of 108 digital specimens. A 3-way analysis of variance (ANOVA) with pairwise comparison and interaction effects was applied to MSA, VV, HD, and DSC for the purpose of determining the best outcomes from Phase 2 from 116 specimens. This was carried out using a statistical software (SPSS, IBM Corp.)

## Supplementary Information

Supplementary Information.

## Data Availability

The data supporting the findings of this study are available within the article and its Supplementary Information file. Source data are provided with this paper.
