# Supplementary material for: Computer-aided design and 3-dimensional artificial/convolutional neural network for digital partial dental crown synthesis and validation
Source: Sci Rep. 2023 Jan 28;13:1561. doi: 10.1038/s41598-023-28442-1 (PMC9884213; doi:10.1038/s41598-023-28442-1)
Supplement: Supplementary file 1 — Supplementary Information. [file 41598_2023_28442_MOESM1_ESM.pdf]

Computer-Aided Design and 3-Dimensional Artificial/Convolutional Neural Network for Digital Partial Dental Crown Synthesis and Validation

SUPPLEMENTARY INFORMATION

Table of Contents

Phase 1: Virtual parameter evaluation outcomes for Intra-oral scanner and Laser scanner 2

Phase 1: Color map of interpoint mismatches, as seen through Hausdorff's Distance analyses 3

Phase 2: Operational commands for Workflow 1 (*3-matics*) 5

Phase 2: Virtual parameter evaluation outcomes for Inlay designs in Workflow 1 7

Phase 2: Virtual parameter evaluation outcomes for Onlay designs in Workflow 1................................. 14

Phase 2: Operational commands for Workflow 2 (*Meshmixer*) 21

Phase 2: Virtual parameter evaluation outcomes for Inlay designs in Workflow 2 23

Phase 2: Virtual parameter evaluation outcomes for Onlay designs in Workflow 2................................. 30

Phase 3: 3-dimensional convolutional neural network sensitivity and specificity 37

Supplementary Figures 38

Supplementary Tables 45

Phase 1: Virtual parameter evaluation outcomes for Intra-oral scanner and Laser scanner

1. **Mesh Surface Area**

Specimen	Intra-oral Scanner	Laser Scanner
1	327.600525	342.248627
2	419.827972	393.203491
3	234.528732	231.195862
4	342.962128	362.116333
5	286.561554	293.953369
6	219.107132	208.216751

2. **Virtual Volume**

Specimen	Intra-oral Scanner	Laser Scanner
1	400.696655	363.118805
2	510.206818	453.532990
3	254.498840	231.579453
4	374.789368	395.742828
5	302.636505	292.229523
6	220.162079	200.031662

3. **Hausdorff's Distance**

Specimen	Intra-oral Scanner Vs Laser Scanner
1	0.097938 ±0.162270
2	0.163047 ±0.230727
3	0.102456 ±0.141916
4	0.041665 ±0.192486
5	-0.025232 ±0.180834
6	0.105432 ±0.149863

4. **Dice Similarity Coefficient**

Specimen	Intra-oral Scanner Vs Laser Scanner
1	0.982488
2	0.900511
3	0.934798
4	0.973759
5	0.937422
6	0.931543

Phase 1: Color map of interpoint mismatches, as seen through Hausdorff's Distance analyses

Specimen	Intraoral scan Vs Laser scan
1	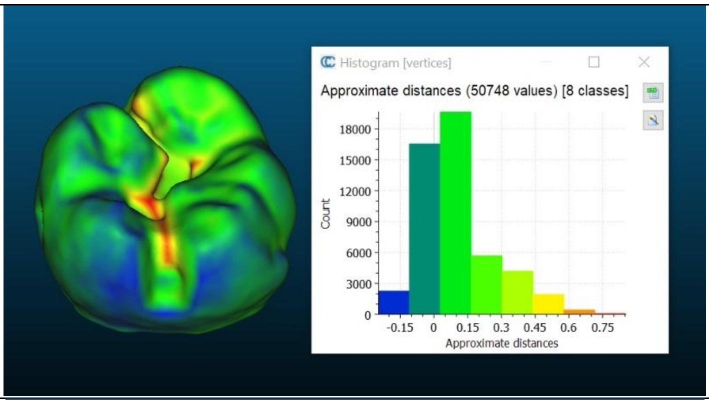
2	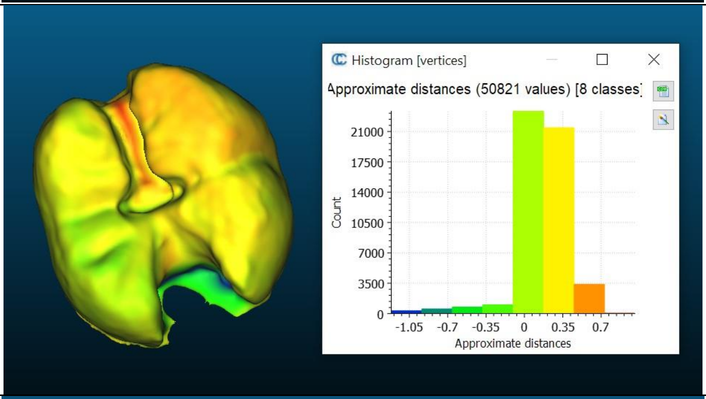
3	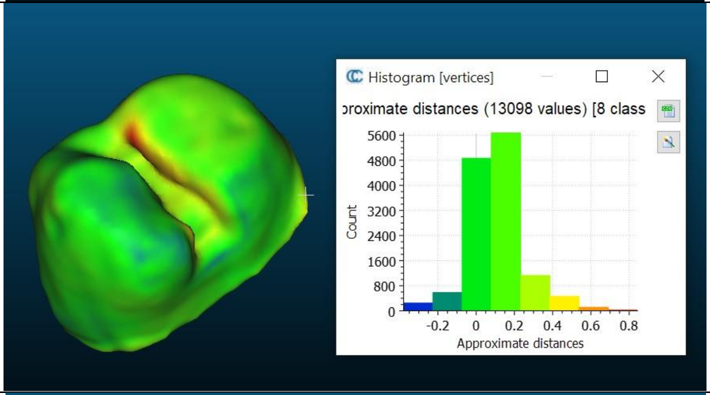
4	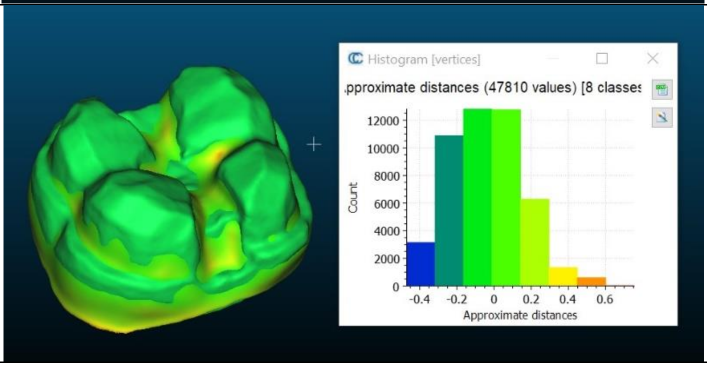

<table>
<tr><td>5</td><td></td><td>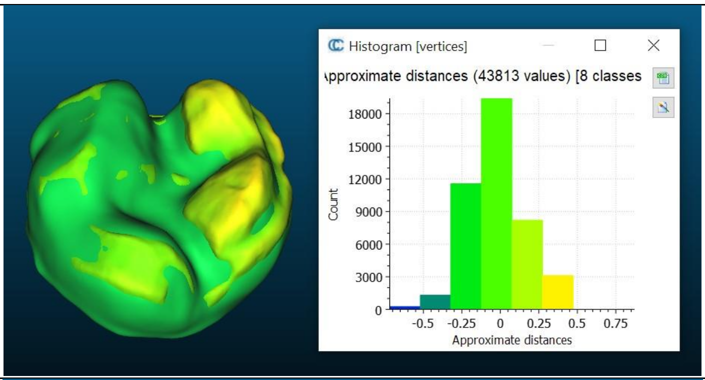
</td><td></td></tr>
<tr><td>6</td><td></td><td>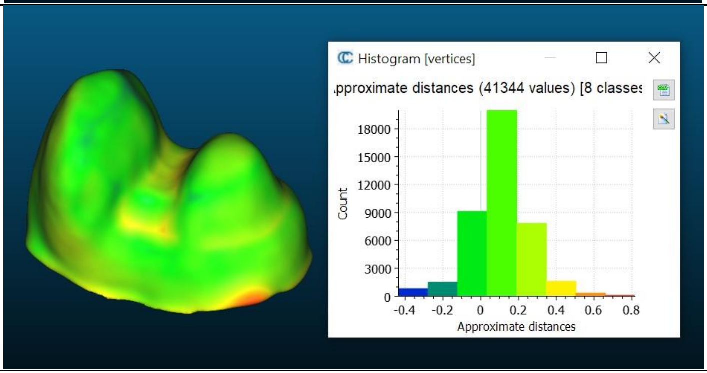
</td><td></td></tr>
</table>

Phase 2: Operational commands for Workflow 1 (*3-matics*)

Inlay

- Open 3matics *> file > import part >* select template > *click ok*
- *Fix > auto fix >* select the imported template > *apply*
- *Analyze > create curvature analysis > apply*
- *Curve > create curve >* create a curve by plotting curve points around the defect > *close curve > select curve > apply*
- *Design > surface construction > select curve > apply*
- *Analysis list > template [Curvature Analysis] > delete > yes*
- *Design > hollow >* select hollow type as 'outside' > *apply*
- *Select the template and entities together > apply*
- *Finish > trim >* plot the trimming points around the reconstructed defect > *apply >* remove the extra areas around the defect > *fix > wrap > select the template and entities together > apply*
- *Select the imported template > delete > yes*
- *Trim >* plot the trimming points around the wrapped template > *apply*
- *File > import part >* select the model > *click ok*
- *Click on mouse right button to hide the 'imported model' > trim >* plot the trimming points to remove any extra portion from the template > *select wrapped template > apply > click on mouse right button to unhide the 'imported model'*
- *Finish > smooth edge >* select the template border > *apply*
- Select the wrapped template > *fix > wrap > apply > click on mouse right button to hide the 'imported model' > file > export > STL >* select the template wrapped as entity > select output directory > *apply*

Onlay

- Open 3matics *> file > import part >* select both the block and onlay template > *click ok*
- *Click on mouse right button to hide the 'imported onlay template' >* select the block template > *click translate/ rotate >* align the block template within the defect > *click on mouse right button to unhide the 'imported onlay template' >* select both the block and onlay template as entity > *design > boolean union > apply*
- *Design > boolean subtraction >* Select both the block and onlay template as 'subtraction entity' > *apply*
- *Finish > trim >* plot the trimming points to remove any extra portion from the template > *apply > smooth edge > apply*
- *Fix > auto fix >* click the subtracted template > *apply*
- *Fix > wrap > apply*

- *File* > *import part* > select the model > *click ok*
- *Select the subtracted without wrapped template* > *delete* > *yes*
- *Select the subtracted wrapped template* > *trim* > plot the trimming points to remove any extra portion from the onlay template > *apply*
- *Click smooth edge* > select the uneven borders as entity > *apply*
- *File* > *export* > *STL* > select the subtracted wrapped onlay template as entity > select output directory > *apply*
- Open Meshmixer > *import the subtracted wrapped onlay template* > *analysis* > *inspector* > *auto repair all* > *done*
- *Edit* > *make solid* > *accurate* > *update* > *accept* > click on mouse right button to delete the unsolid STL template file
- *Sculpt* > select the *robust smooth brush tool* to smooth out the uneven portion from the reconstructed onlay template
- *export as STL format*

Phase 2: Virtual parameter evaluation outcomes for Inlay designs in Workflow 1

1. Mesh Surface Area

Samples	Operator 1	Operator 2
1	186.512756	144.316422
2	140.311783	130.574417
3	140.617798	141.272156
4	188.360504	149.632202
5	176.387329	147.777008
6	200.940720	169.872528
7	201.436844	165.147079
8	196.707825	169.939682
9	145.364410	159.147202
10	174.906113	153.988525
11	52.135353	66.567986
12	56.126816	70.996414
13	53.823254	71.575768
14	50.548229	73.959114
15	62.782219	--------------

2. Virtual Volume

Samples	Operator 1	Operator 2
1	43.007740	40.222443
2	42.361702	44.959412
3	41.420044	45.908550
4	46.427631	46.056927
5	48.877945	44.704880
6	55.985210	50.303127
7	51.113541	55.757034
8	43.040730	49.315315
9	41.912964	45.974125
10	41.551579	42.975426
11	20.546768	19.775017
12	19.248747	20.511940
13	19.606424	20.112736
14	19.690113	21.693691
15	21.406635	--------------

3. **Hausdorff's Distance**

Samples	Operator 1	Operator 2
1	-0.099713 ±0.582039	-0.143359 ±0.594401
2	-0.154024 ±0.607225	-0.174311 ±0.619415
3	-0.162578 ±0.596869	-0.178337 ±0.603924
4	-0.103928 ±0.572541	-0.167632 ±0.598728
5	-0.122722 ±0.592649	-0.156820 ±0.611431
6	-0.270936 ±0.633483	-0.321239 ±0.628782
7	-0.279524 ±0.619985	-0.265048 ±0.414909
8	-0.281080 ±0.569685	-0.339844 ±0.581191
9	-0.398268 ±0.576353	-0.359690 ±0.566294
10	-0.326523 ±0.553245	-0.355042 ±0.551709
11	-0.284103 ±0.547951	-0.216453 ±0.523173
12	-0.275818 ±0.544664	-0.209127 ±0.539585
13	-0.284476 ±0.543860	-0.205154 ±0.526702
14	-0.308967 ±0.538112	-0.188476 ±0.541088
15	-0.257934 ±0.527669	----------------------------

a) Comparison made against virtually scanned and conventionally made Inlays
b) Hausdorff's distance measured with onlays attached to the samples, to mitigate oversampling influenced inconsistencies

4. Color map of Hausdorff's distance

Specimen	Operator 1	Operator 2
1	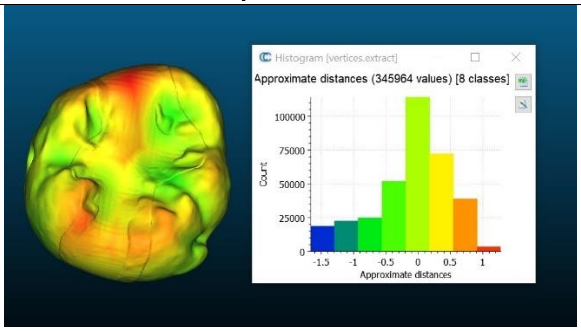	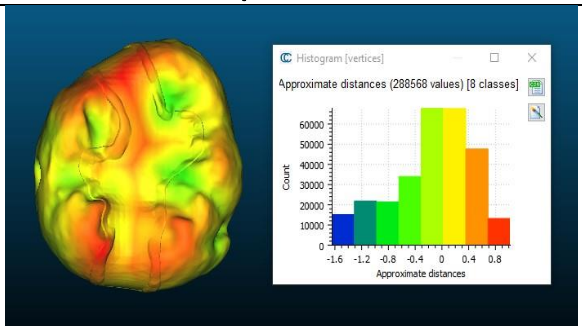
2	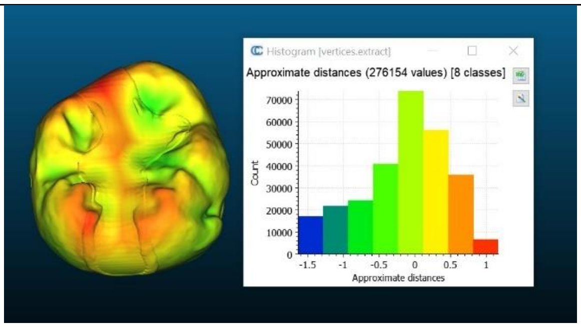	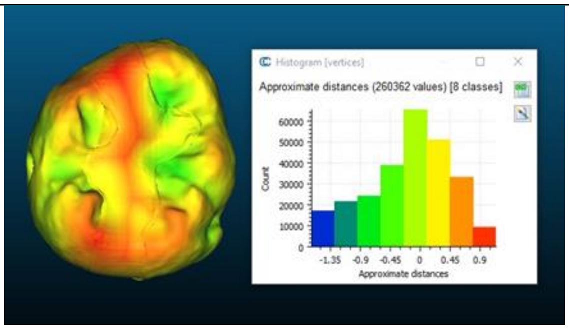
3	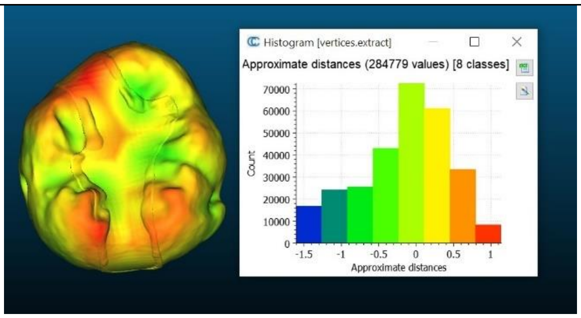	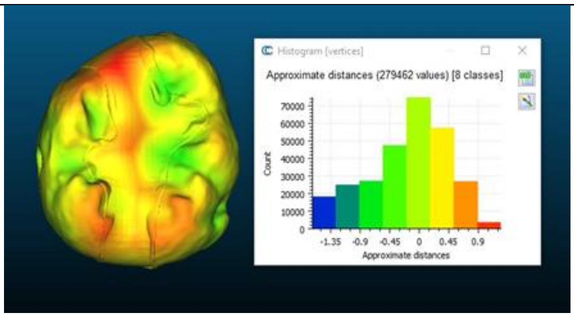
4	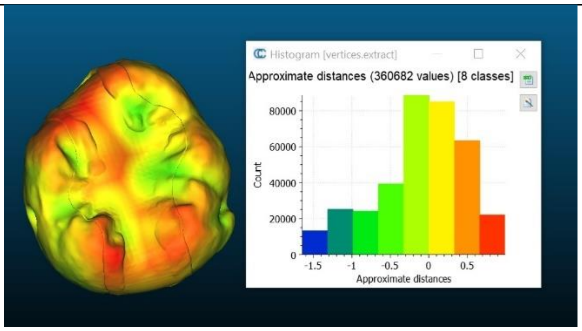	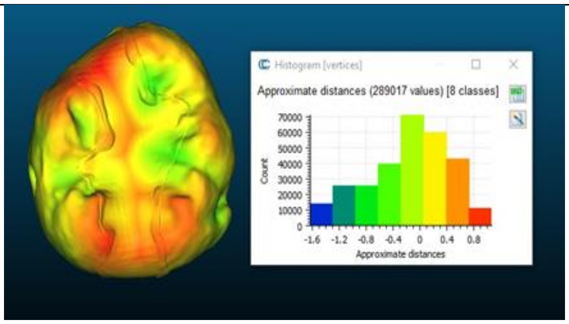

<table>
<tr><td>5</td><td>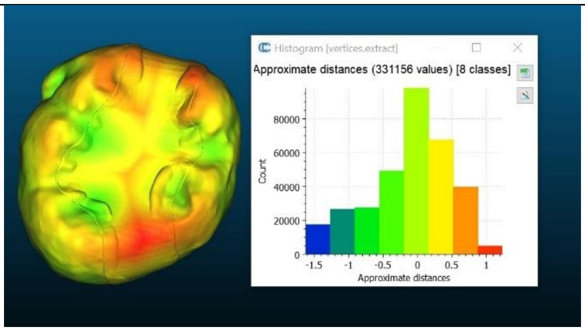
</td><td>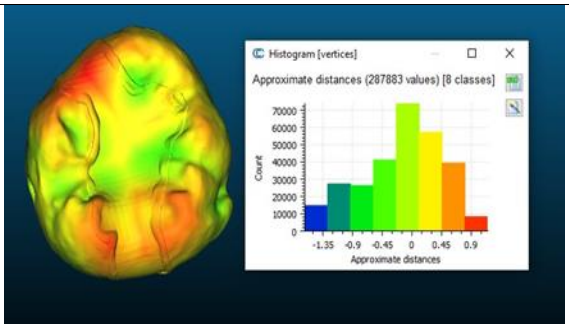
</td></tr>
<tr><td>6</td><td>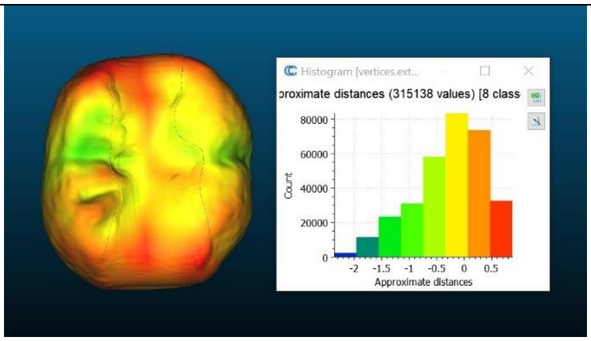
</td><td>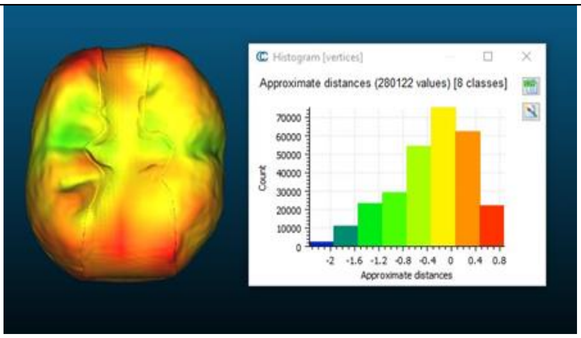
</td></tr>
<tr><td>7</td><td>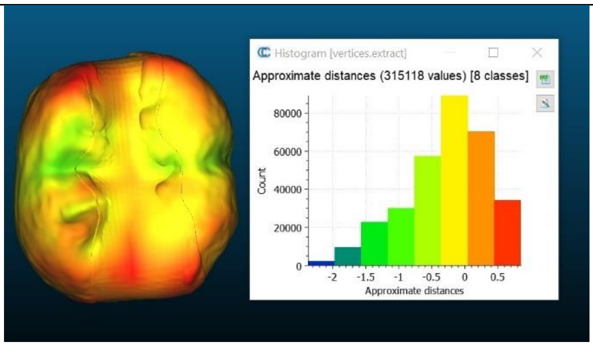
</td><td>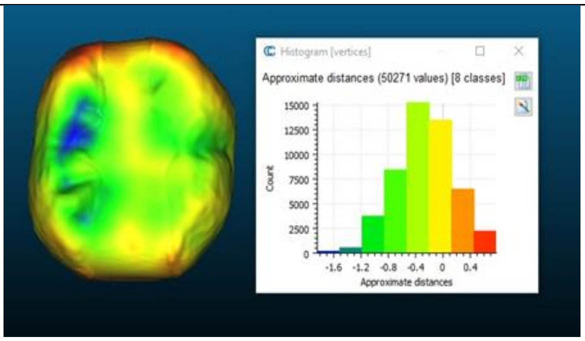
</td></tr>
<tr><td>8</td><td>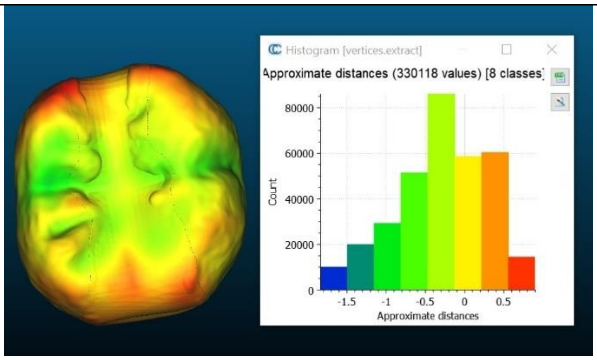
</td><td>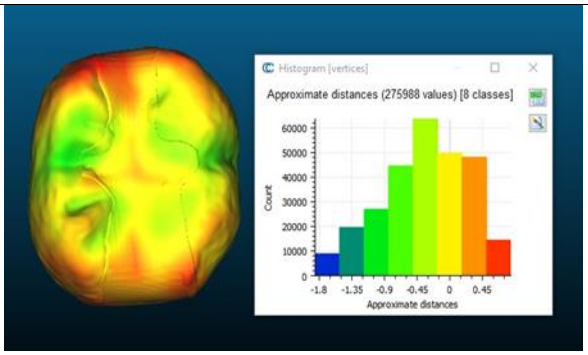
</td></tr>
</table>

9	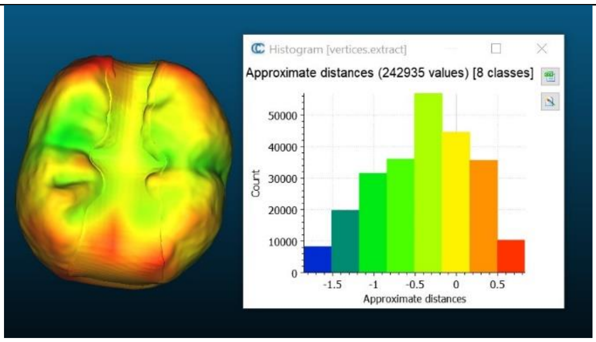Histogram [vertices.extract]<br>Approximate distances (242935 values) [8 classes]<br>50000 40000 30000 20000 10000 0<br>Count<br>-1.5 -1 -0.5 0 0.5<br>Approximate distances	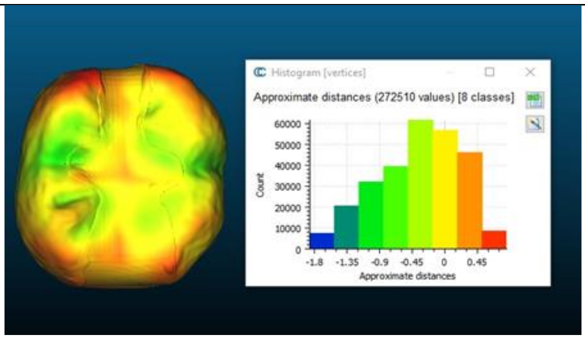Histogram [vertices]<br>Approximate distances (272510 values) [8 classes]<br>60000 50000 40000 30000 20000 10000 0<br>Count<br>-1.8 -1.35 -0.9 -0.45 0 0.45<br>Approximate distances
10	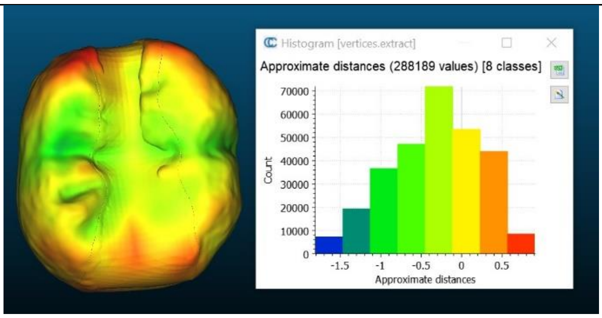Histogram [vertices.extract]<br>Approximate distances (288189 values) [8 classes]<br>70000 60000 50000 40000 30000 20000 10000 0<br>Count<br>-1.5 -1 -0.5 0 0.5<br>Approximate distances	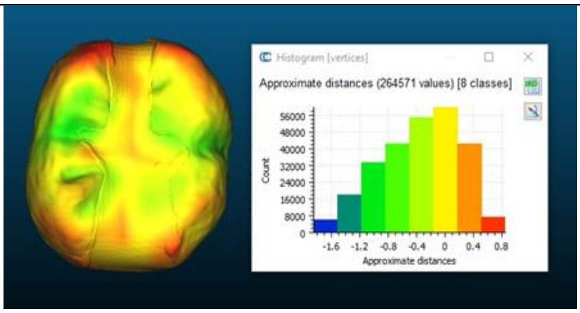Histogram [vertices]<br>Approximate distances (264571 values) [8 classes]<br>56000 48000 40000 32000 24000 16000 8000 0<br>Count<br>-1.6 -1.2 -0.8 -0.4 0 0.4 0.8<br>Approximate distances
11	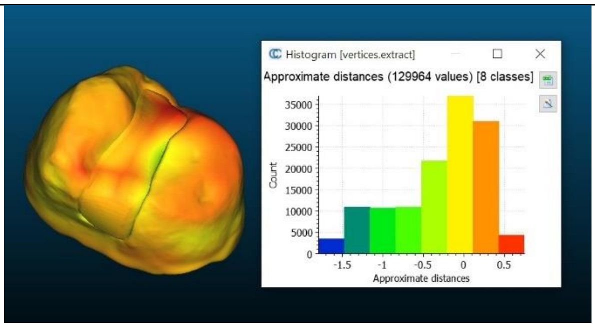Histogram [vertices.extract]<br>Approximate distances (129964 values) [8 classes]<br>35000 30000 25000 20000 15000 10000 5000 0<br>Count<br>-1.5 -1 -0.5 0 0.5<br>Approximate distances	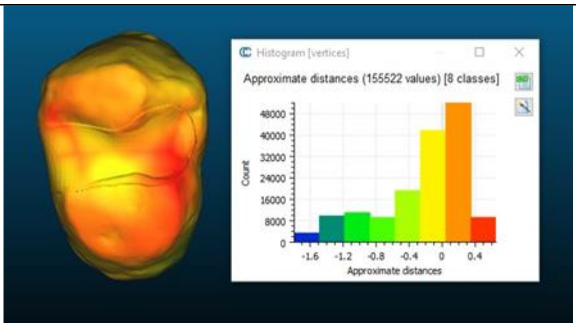Histogram [vertices]<br>Approximate distances (155522 values) [8 classes]<br>48000 40000 32000 24000 16000 8000 0<br>Count<br>-1.6 -1.2 -0.8 -0.4 0 0.4<br>Approximate distances
12	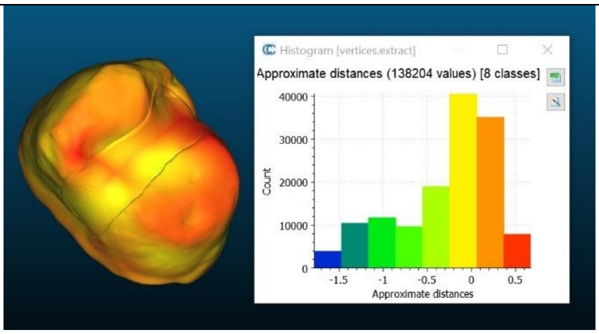Histogram [vertices.extract]<br>Approximate distances (138204 values) [8 classes]<br>40000 30000 20000 10000 0<br>Count<br>-1.5 -1 -0.5 0 0.5<br>Approximate distances	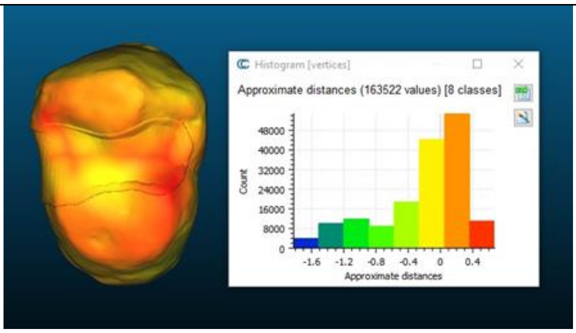Histogram [vertices]<br>Approximate distances (163522 values) [8 classes]<br>48000 40000 32000 24000 16000 8000 0<br>Count<br>-1.6 -1.2 -0.8 -0.4 0 0.4<br>Approximate distances

<table>
<tr>
<td>13</td>
<td>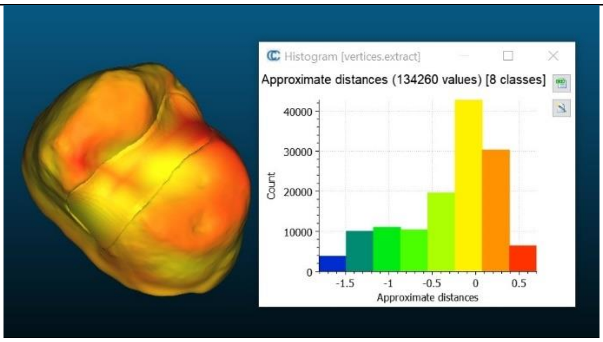
</td>
<td>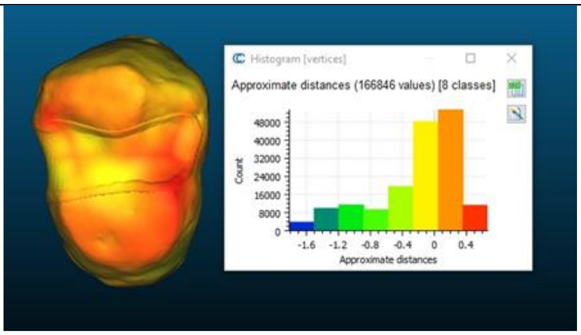
</td>
</tr>
<tr>
<td>14</td>
<td>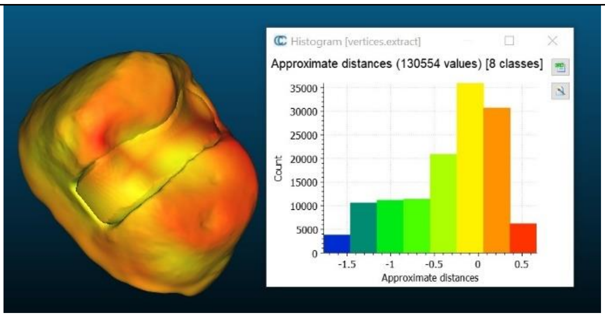
</td>
<td>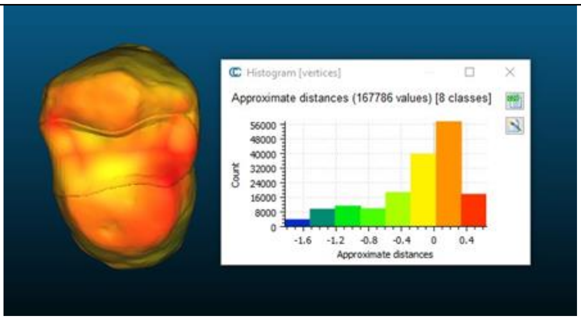
</td>
</tr>
<tr>
<td>15</td>
<td>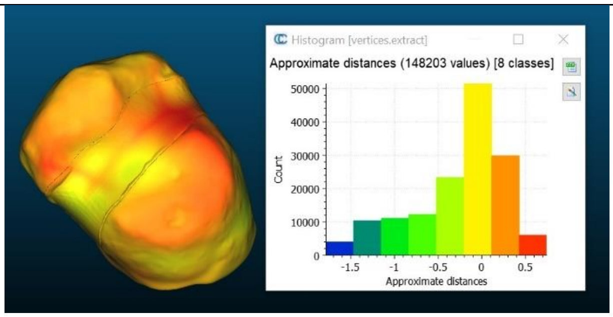
</td>
<td>---------------------</td>
</tr>
</table>

5. Dice Similarity Coefficient

Samples	Operator 1	Operator 2
1	0.882399	0.882935
2	0.885365	0.880513
3	0.879976	0.878344
4	0.873307	0.875692
5	0.878770	0.871556
6	0.810718	0.810537
7	0.811733	0.859077
8	0.823478	0.821768
9	0.819397	0.821604
10	0.823404	0.823241
11	0.899681	0.899018
12	0.896448	0.899380
13	0.897757	0.897433
14	0.892376	0.896851
15	0.891767	-------------

a) Comparison made against virtually scanned and conventionally made Inlays
b) Hausdorff's distance measured with onlays attached to the samples, to mitigate oversampling influenced inconsistencies

Phase 2: Virtual parameter evaluation outcomes for Onlay designs in Workflow 1

1. Mesh Surface Area

Samples	Operator 1	Operator 2
1	416.101013	429.512756
2	429.105652	429.522369
3	427.752441	429.922821
4	422.481384	426.848480
5	431.557037	427.603333
6	306.987030	304.636597
7	298.155640	302.355591
8	291.782257	303.721588
9	291.741699	292.526520
10	292.376129	299.231842
11	126.483856	154.739441
12	123.609154	145.099319
13	144.313873	147.915985
14	144.852097	142.865311
15	141.997452	141.521683

2. Virtual Volume

Samples	Operator 1	Operator 2
1	266.334625	267.945709
2	279.134735	267.168030
3	280.999176	271.075562
4	272.959381	274.373901
5	271.747314	267.328400
6	168.008575	177.510956
7	147.456360	170.604324
8	145.857239	170.675095
9	143.538559	160.532059
10	143.447021	164.123215
11	47.163601	63.232418
12	43.129753	58.611378
13	66.357841	59.972904
14	66.397820	59.054153
15	62.193859	57.232430

3. Hausdorff’s Distance

Samples	Operator 1	Operator 2
1	-0.354353 ±0.731484	-0.343511 ±0.736753
2	-0.337710 ±0.739113	-0.244376 ±0.705227
3	-0.341124 ±0.735458	-0.331802 ±0.716778
4	-0.350489 ±0.724673	-0.328679 ±0.720684
5	-0.338180 ±0.729628	-0.339855 ±0.712887
6	-0.094843 ±0.174660	-0.318753 ±0.598738
7	-0.219360 ±0.325744	-0.333626 ±0.598629
8	-0.239727 ±0.334353	-0.333108 ±0.594664
9	-0.234194 ±0.308532	-0.339256 ±0.589377
10	-0.247104 ±0.337820	-0.104982 ±0.177127
11	-0.219281 ±0.470172	-0.197792 ±0.512537
12	-0.234025 ±0.469331	-0.217895 ±0.509120
13	-0.247227 ±0.520365	-0.195837 ±0.523231
14	-0.235327 ±0.513909	-0.236994 ±0.512880
15	-0.224722 ±0.515644	-0.223254 ±0.513661

c) Comparison made against virtually scanned and conventionally made Onlays
d) Hausdorff’s distance measured with onlays attached to the samples, to mitigate oversampling influenced inconsistencies

4. Color map of Hausdorff's distance

<table>
<tr><th>Specimen</th><th>Operator 1</th><th>Operator 2</th></tr>
<tr><td>1</td><td>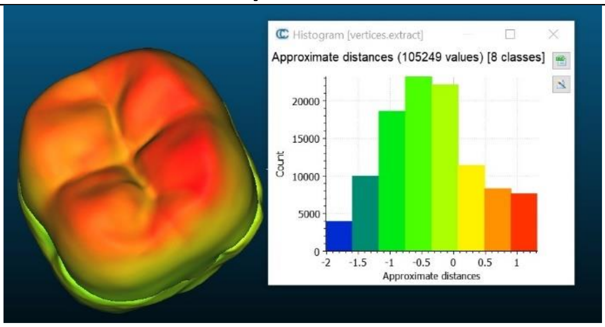
</td><td>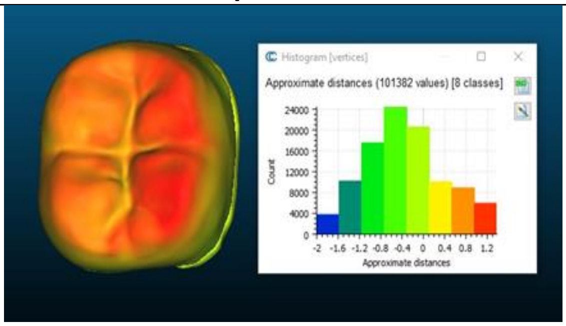
</td></tr>
<tr><td>2</td><td>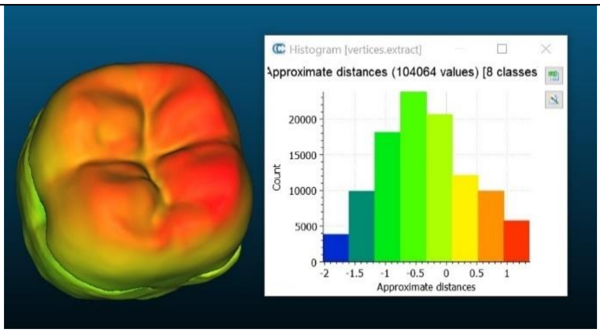
</td><td>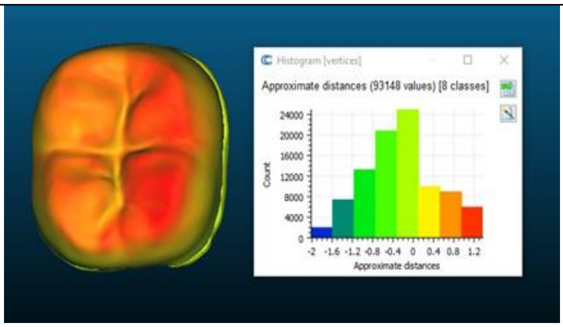
</td></tr>
<tr><td>3</td><td>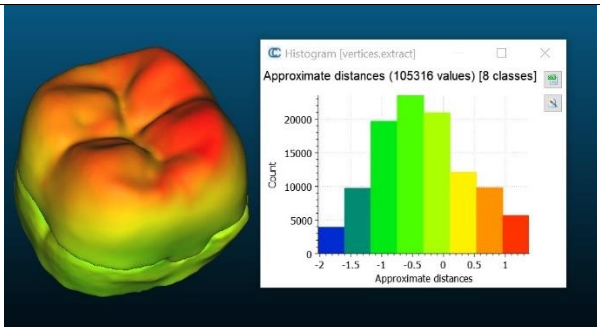
</td><td>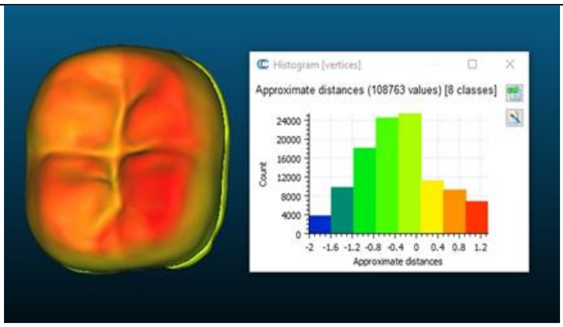
</td></tr>
<tr><td>4</td><td>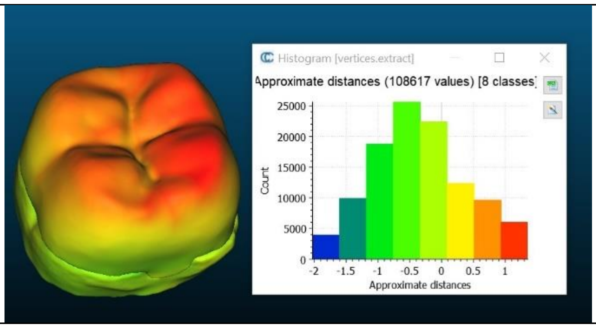
</td><td>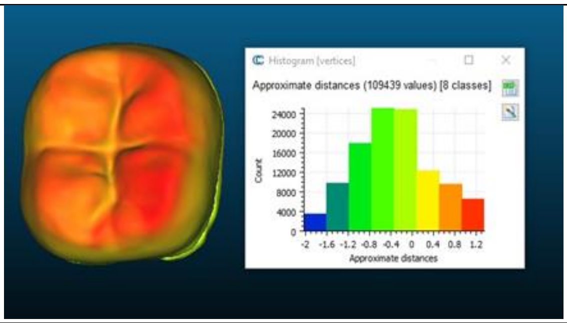
</td></tr>
</table>

5	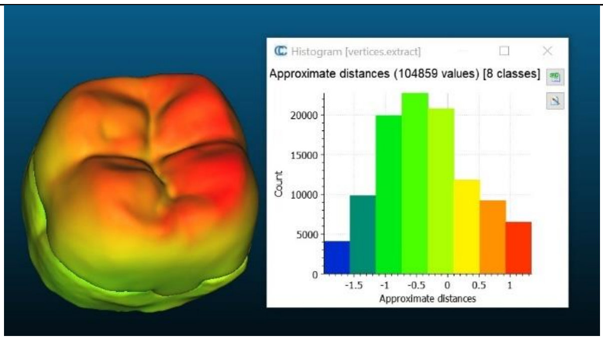Histogram [vertices.extract]<br>Approximate distances (104859 values) [8 classes]<br>Count<br>Approximate distances	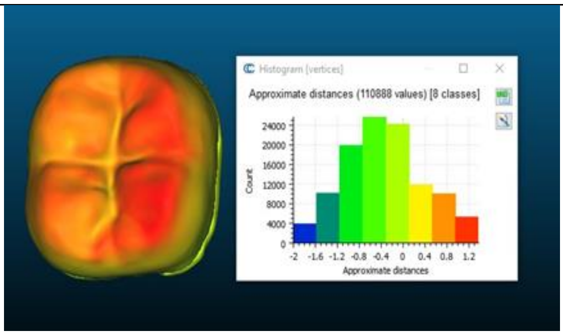Histogram [vertices]<br>Approximate distances (110888 values) [8 classes]<br>Count<br>Approximate distances
6	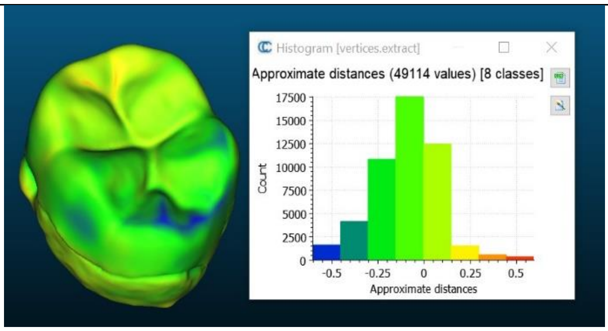Histogram [vertices.extract]<br>Approximate distances (49114 values) [8 classes]<br>Count<br>Approximate distances	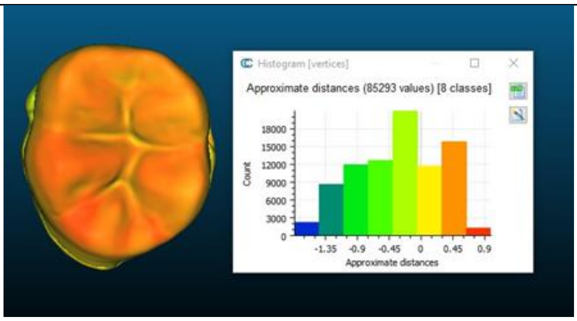Histogram [vertices]<br>Approximate distances (85293 values) [8 classes]<br>Count<br>Approximate distances
7	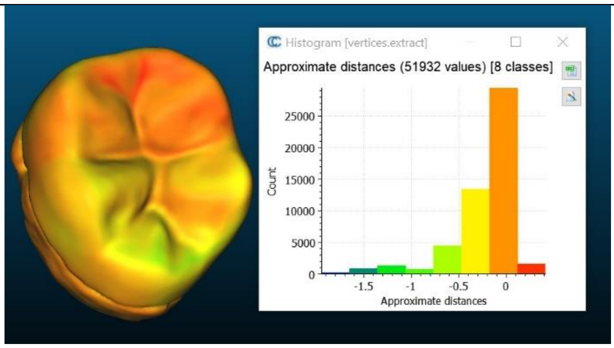Histogram [vertices.extract]<br>Approximate distances (51932 values) [8 classes]<br>Count<br>Approximate distances	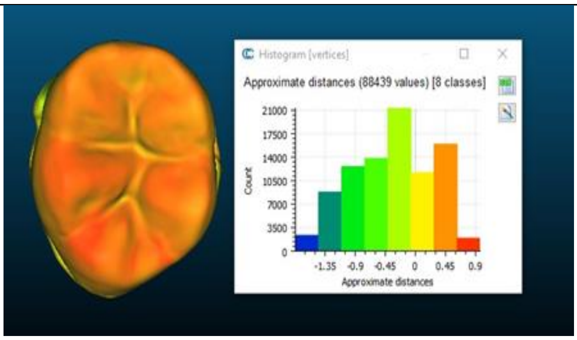Histogram [vertices]<br>Approximate distances (88439 values) [8 classes]<br>Count<br>Approximate distances
8	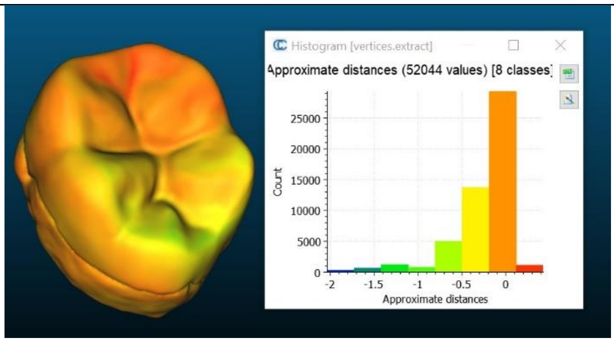Histogram [vertices.extract]<br>Approximate distances (52044 values) [8 classes]<br>Count<br>Approximate distances	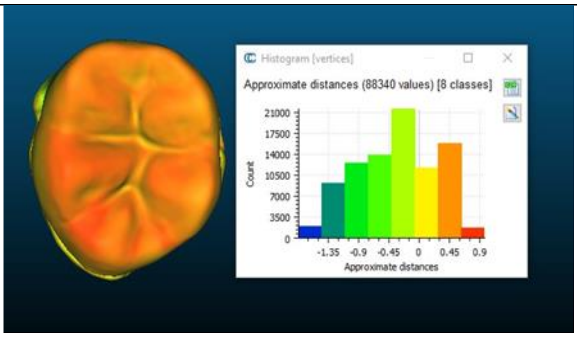Histogram [vertices]<br>Approximate distances (88340 values) [8 classes]<br>Count<br>Approximate distances

9	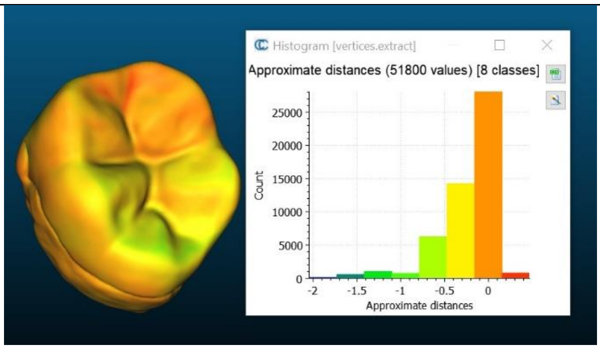Histogram [vertices.extract]<br>Approximate distances (51800 values) [8 classes]<br>Count<br>25000 20000 15000 10000 5000 0<br>-2 -1.5 -1 -0.5 0<br>Approximate distances	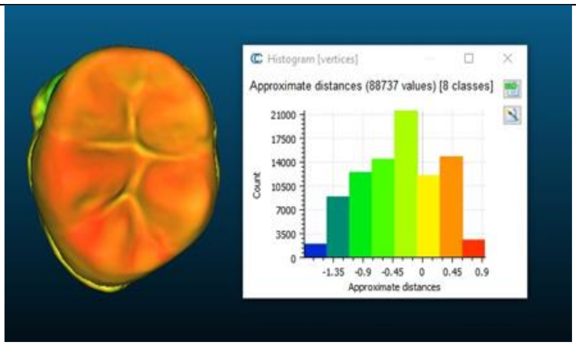Histogram [vertices]<br>Approximate distances (88737 values) [8 classes]<br>Count<br>21000 17500 14000 10500 7000 3500 0<br>-1.35 -0.9 -0.45 0 0.45 0.9<br>Approximate distances
10	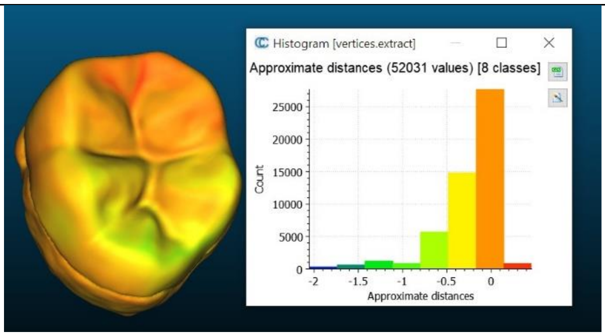Histogram [vertices.extract]<br>Approximate distances (52031 values) [8 classes]<br>Count<br>25000 20000 15000 10000 5000 0<br>-2 -1.5 -1 -0.5 0<br>Approximate distances	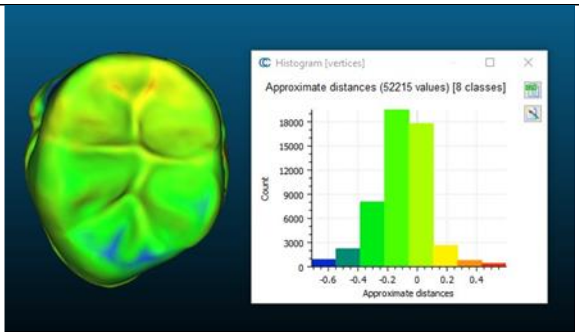Histogram [vertices]<br>Approximate distances (52215 values) [8 classes]<br>Count<br>18000 15000 12000 9000 6000 3000 0<br>-0.6 -0.4 -0.2 0 0.2 0.4<br>Approximate distances
11	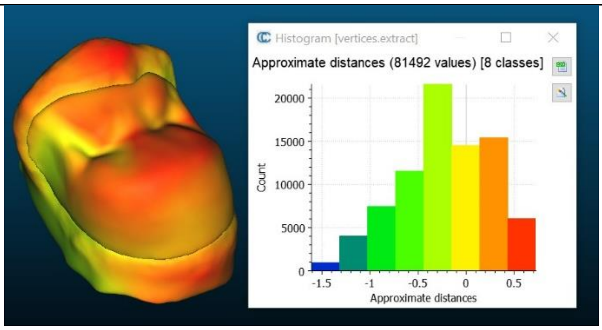Histogram [vertices.extract]<br>Approximate distances (81492 values) [8 classes]<br>Count<br>20000 15000 10000 5000 0<br>-1.5 -1 -0.5 0 0.5<br>Approximate distances	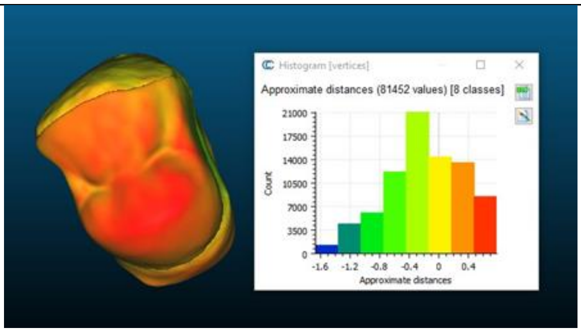Histogram [vertices]<br>Approximate distances (81452 values) [8 classes]<br>Count<br>21000 17500 14000 10500 7000 3500 0<br>-1.6 -1.2 -0.8 -0.4 0 0.4<br>Approximate distances
12	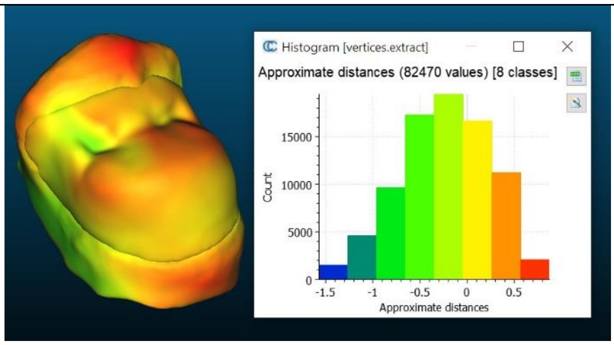Histogram [vertices.extract]<br>Approximate distances (82470 values) [8 classes]<br>Count<br>15000 10000 5000 0<br>-1.5 -1 -0.5 0 0.5<br>Approximate distances	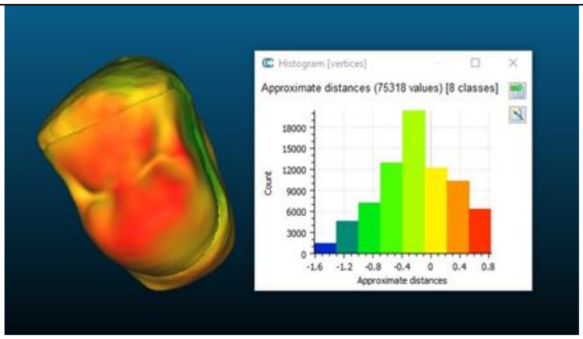Histogram [vertices]<br>Approximate distances (75318 values) [8 classes]<br>Count<br>18000 15000 12000 9000 6000 3000 0<br>-1.6 -1.2 -0.8 -0.4 0 0.4 0.8<br>Approximate distances

13	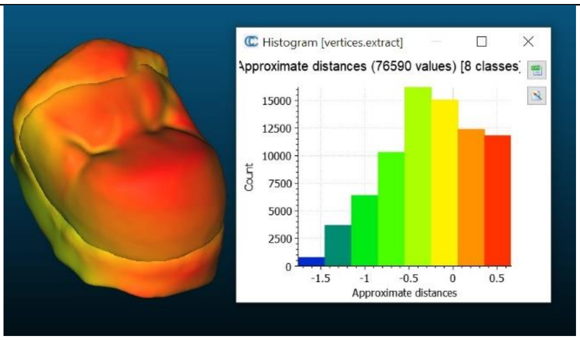Histogram [vertices.extract]<br>Approximate distances (76590 values) [8 classes]<br>Count<br>Approximate distances	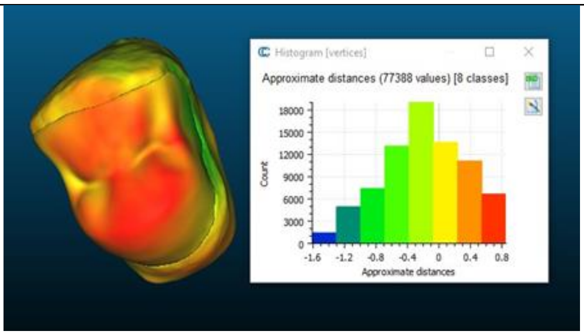Histogram [vertices]<br>Approximate distances (77388 values) [8 classes]<br>Count<br>Approximate distances
14	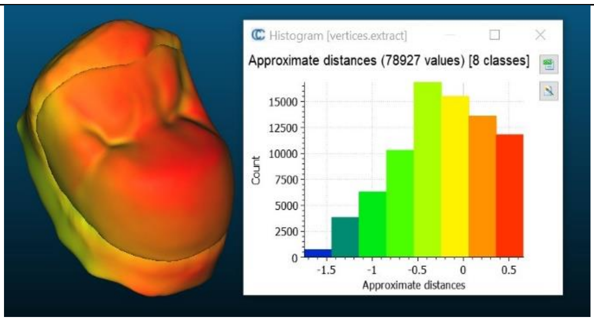Histogram [vertices.extract]<br>Approximate distances (78927 values) [8 classes]<br>Count<br>Approximate distances	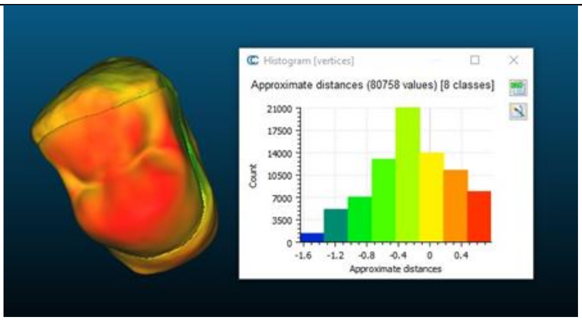Histogram [vertices]<br>Approximate distances (80758 values) [8 classes]<br>Count<br>Approximate distances
15	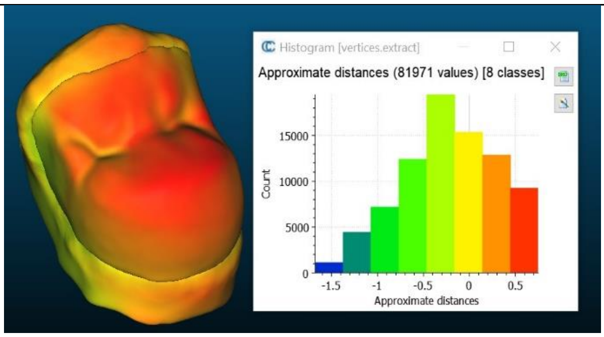Histogram [vertices.extract]<br>Approximate distances (81971 values) [8 classes]<br>Count<br>Approximate distances	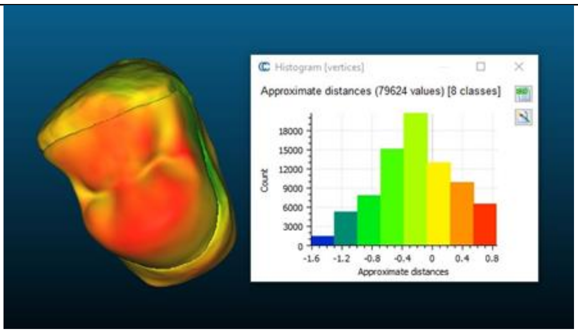Histogram [vertices]<br>Approximate distances (79624 values) [8 classes]<br>Count<br>Approximate distances

5. Dice Similarity Coefficient

Samples	Operator 1	Operator 2
1	0.836770	0.818140
2	0.828649	0.833628
3	0.827020	0.829545
4	0.834012	0.824578
5	0.829519	0.818872
6	0.943911	0.853179
7	0.930426	0.842947
8	0.923915	0.847213
9	0.921245	0.841204
10	0.921626	0.940321
11	0.828881	0.855908
12	0.827747	0.837395
13	0.840568	0.841065
14	0.837845	0.846386
15	0.850172	0.841248

a) Comparison made against virtually scanned and conventionally made Onlays
b) Hausdorff's distance measured with onlays attached to the samples, to mitigate oversampling influenced inconsistencies

Phase 2: Operational commands for Workflow 2 (*Meshmixer*)

Inlay

- Open the STL file of tooth scan with Autodesk Meshmixer
- Select the block template > drag and drop to the Autodesk Meshmixer> *append > shift position: no*.
- *Edit* > *world frame* > manually adjust the block to the defect > *accept*
- *Ctrl* (select block template + inlay template) > *Boolean difference > accept > edit > make solid > solid type: accurate > accept*
- *Object browser: block template. stl > delete*
- *Select* (manually mark the excess peripherals) > *delete*
- *Analysis > inspector > auto repair all > done*
- *Edit > make solid > solid type: accurate > update > accept*
- Drag and drop the STL file of inlay tooth scan to Autodesk Meshmixer > append > shift position: no.
- *Object browser: block template. stl (solid) (solid) > sculpt > brushes > shrink smooth* (manually smoothen the sharp edges)
- *brushes > inflate* (manually make the grooves of occlusal surface according to the tooth anatomy) *> robust smooth* (manually smooth) > *shrink smooth* (manually smooth) > *drag* (manually cover the defects if any area remains uncovered) > *Flatten* (manually flatten the margins where needed)
- *export as STL format*

Onlay

- Open the STL file of tooth scan with Autodesk Meshmixer
- *Select* the block template > drag and drop to the Autodesk Meshmixer> *append > shift position: no*.
- *Edit* > *world frame* > manually adjust the block to the defect > *accept*
- Select onlay template > drag and drop to Autodesk Meshmixer > *append > shift position: no*
- *Ctrl* (select block template + onlay template) > *combine > edit > make solid > solid type: accurate > update > accept*
- *Object browser: block template stl > delete*
- *Object browser: ctrl [select onlay. stl + block template. stl (solid)] > Boolean difference > accept*
- *Analysis > inspector > auto repair all > edit > make solid > solid type: accurate > update > accept*

- Drag and drop the STL file of tooth scan to Autodesk Meshmixer > *append* > *shift position: no*
- *Object browser: block template. stl (solid) (solid*) > *select* > manually mark the excess peripherals > *delete*
- *Analysis > inspector > auto repair all > edit > make solid > solid type: accurate > update > accept*
- *Object browser: block template. stl (solid) (solid) > delete*
- *Object browser: block template (solid) (solid) (solid) > sculpt > brushes > flatten* (manually flatten the edges) > *shrink smooth* (manually smoothen the irregularities) *> robust smooth*
- *Object browser: onlay. stl (set as target) > block template. stl (solid) (solid) (solid) > sculpt > brushes > shrink smooth* (manually smoothen the excesses)
- *Object browser: onlay. stl (hide object) > sculpt > brushes > robust smooth* (manually smooth the irregular edges) > show object (onlay. stl) > *sculpt > brushes > drag* (manually cover the defects if any area remains uncovered)
- *export* as *STL format*

Phase 2: Virtual parameter evaluation outcomes for Inlay designs in Workflow 2

1. Mesh Surface Area

Samples	Operator 1	Operator 2
1	95.384743	95.599907
2	104.588898	99.280701
3	103.152351	100.028564
4	105.331421	105.556633
5	105.090652	103.551941
6	114.674393	115.027374
7	109.005348	117.420898
8	109.116737	113.890030
9	106.831161	101.972191
10	106.431557	105.669388
11	51.391506	49.402653
12	50.423153	47.921539
13	50.897137	49.071480
14	51.858192	49.311707
15	56.229607	55.402107

2. Virtual Volume

Samples	Operator 1	Operator 2
1	38.156460	37.548470
2	40.243011	37.432858
3	43.555904	39.762962
4	42.160534	41.008389
5	46.490730	41.531620
6	48.814018	48.373783
7	43.999088	49.986832
8	42.375202	43.157249
9	39.120743	35.455460
10	36.668194	36.054703
11	18.828712	19.843365
12	19.356865	18.982983
13	18.828136	17.542906
14	17.999161	17.097883
15	21.252043	19.983858

3. Hausdorff's Distance

Samples	Operator 1	Operator 2
1	-0.046352 ±0.208370	-0.191179 ±0.521602
2	-0.049777 ±0.210664	-0.185989 ±0.519171
3	-0.041633 ±0.214145	-0.192801 ±0.516250
4	-0.055994 ±0.216792	-0.187743 ±0.524317
5	-0.045923 ±0.213023	-0.194326 ±0.529987
6	-0.301226 ±0.434545	-0.402317 ±0.628927
7	-0.305183 ±0.433800	-0.382326 ±0.644880
8	-0.298800 ±0.440940	-0.370403 ±0.564651
9	-0.305765 ±0.431150	-0.411835 ±0.572923
10	-0.310213 ±0.440414	-0.411713 ±0.558276
11	-0.077779 ±0.139746	-0.312563 ±0.531053
12	-0.081451 ±0.139149	-0.337719 ±0.539888
13	-0.080388 ±0.137801	-0.327381 ±0.515654
14	-0.088769 ±0.157490	-0.308788 ±0.499512
15	-0.080655 ±0.140072	-0.270326 ±0.491395

c) Comparison made against virtually scanned and conventionally made Inlays
d) Hausdorff's distance measured with onlays attached to the samples, to mitigate oversampling influenced inconsistencies

4. Color map of Hausdorff's distance

Specimen	Operator 1	Operator 2
1	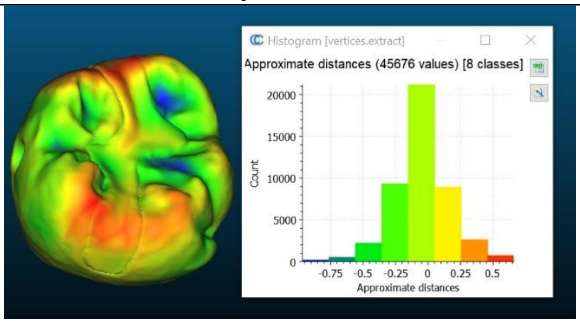	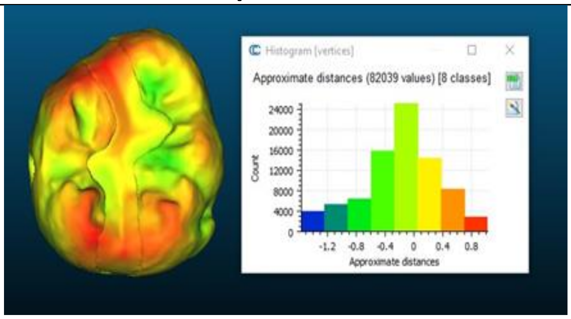
2	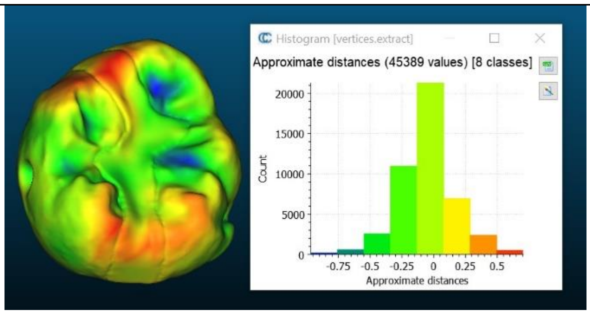	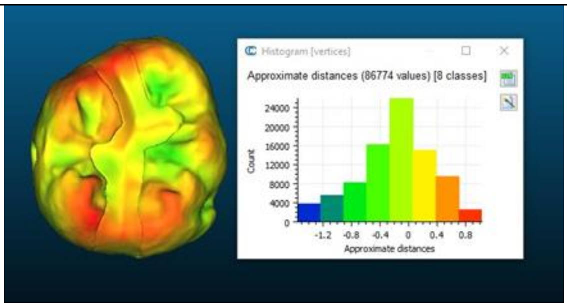
3	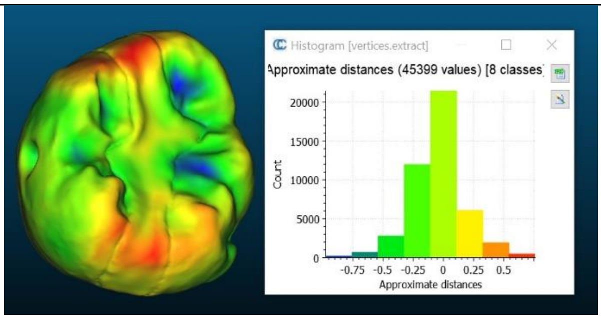	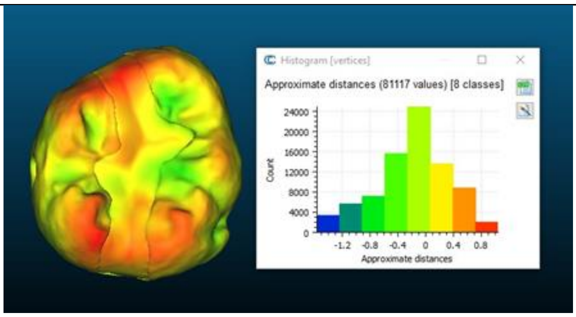
4	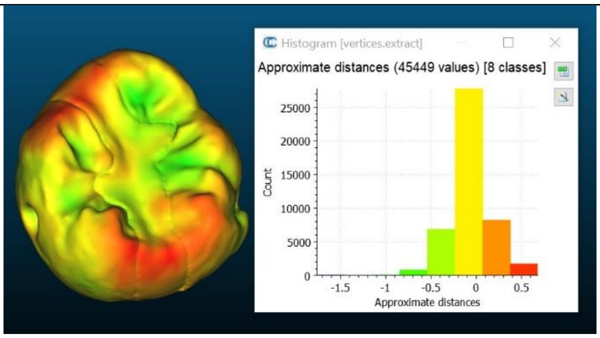	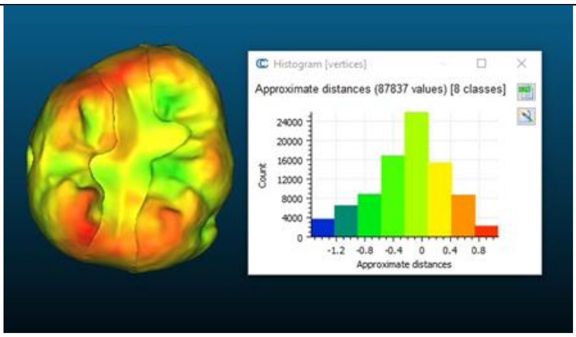

5	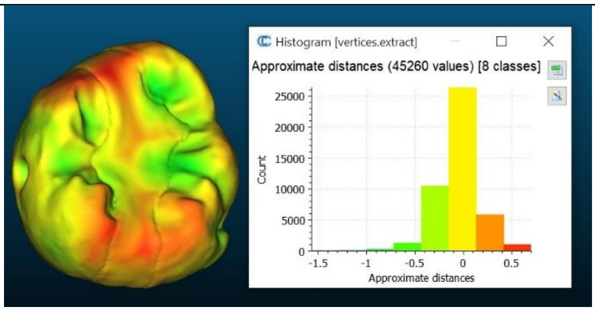Histogram [vertices.extract]<br>Approximate distances (45260 values) [8 classes]<br>Count<br>Approximate distances	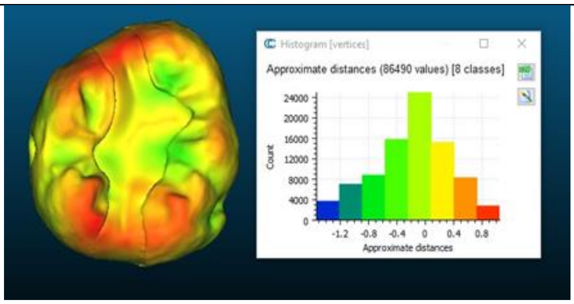Histogram [vertices]<br>Approximate distances (86490 values) [8 classes]<br>Count<br>Approximate distances
6	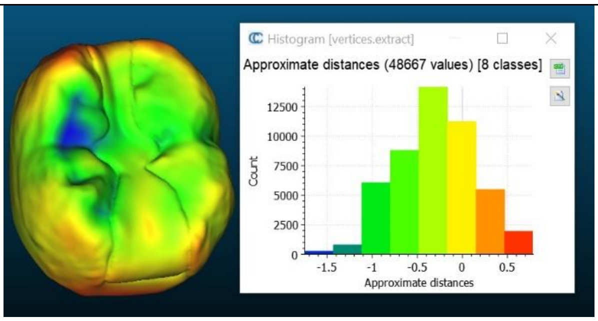Histogram [vertices.extract]<br>Approximate distances (48667 values) [8 classes]<br>Count<br>Approximate distances	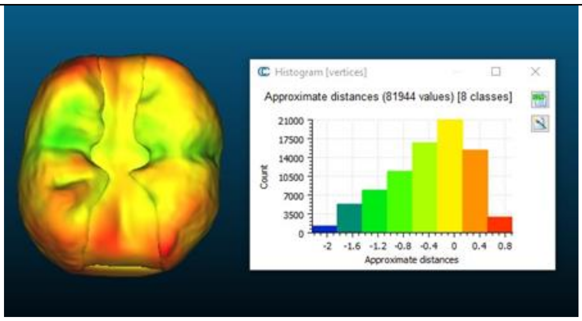Histogram [vertices]<br>Approximate distances (81944 values) [8 classes]<br>Count<br>Approximate distances
7	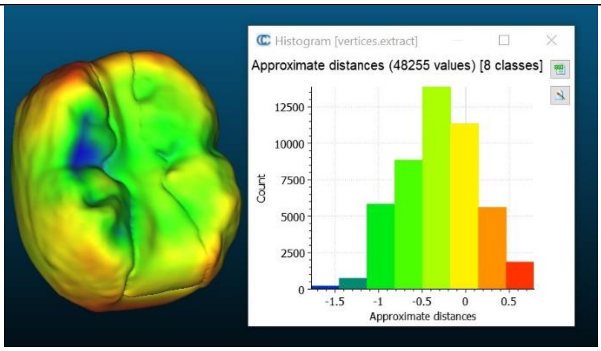Histogram [vertices.extract]<br>Approximate distances (48255 values) [8 classes]<br>Count<br>Approximate distances	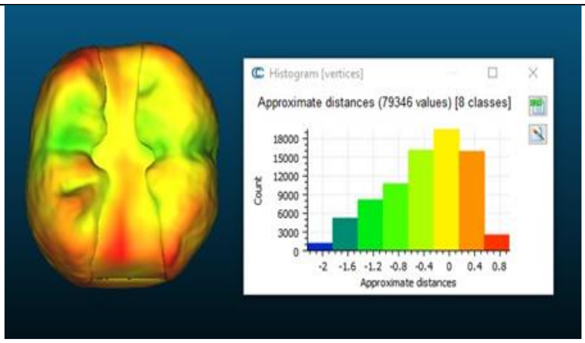Histogram [vertices]<br>Approximate distances (79346 values) [8 classes]<br>Count<br>Approximate distances
8	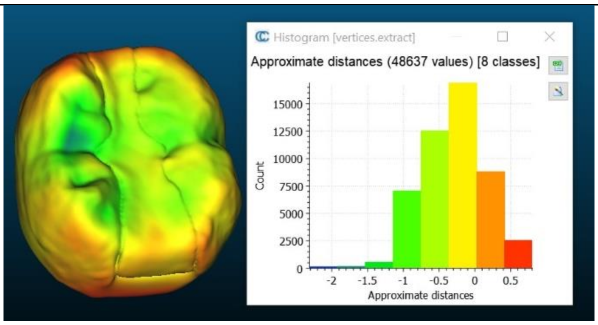Histogram [vertices.extract]<br>Approximate distances (48637 values) [8 classes]<br>Count<br>Approximate distances	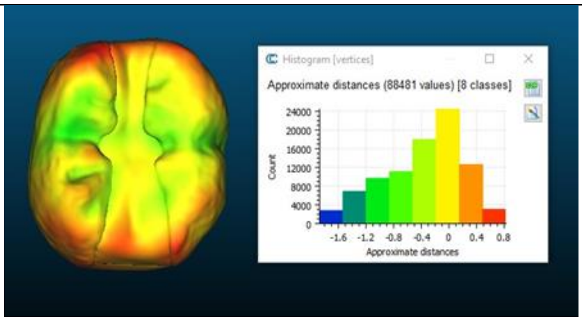Histogram [vertices]<br>Approximate distances (88481 values) [8 classes]<br>Count<br>Approximate distances

9	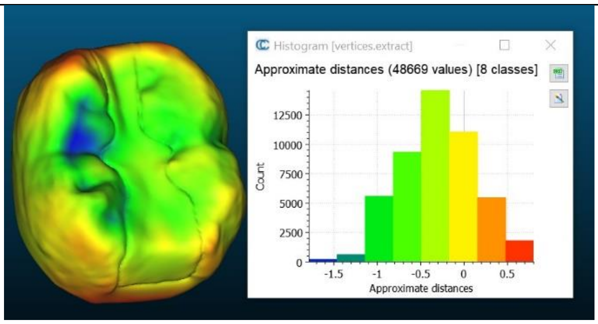Histogram [vertices.extract]<br>Approximate distances (48669 values) [8 classes]<br>Count<br>12500 10000 7500 5000 2500 0<br>-1.5 -1 -0.5 0 0.5<br>Approximate distances	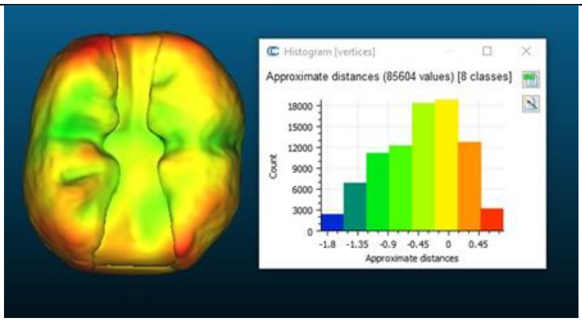Histogram [vertices]<br>Approximate distances (85604 values) [8 classes]<br>Count<br>18000 15000 12000 9000 6000 3000 0<br>-1.8 -1.35 -0.9 -0.45 0 0.45<br>Approximate distances
10	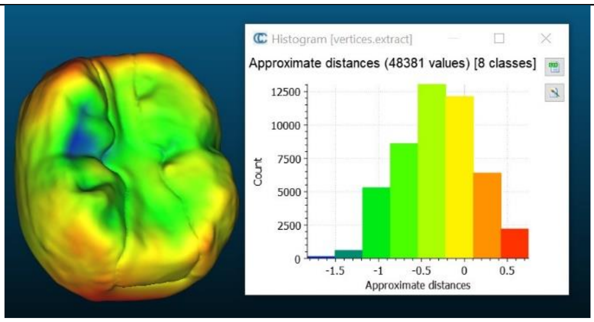Histogram [vertices.extract]<br>Approximate distances (48381 values) [8 classes]<br>Count<br>12500 10000 7500 5000 2500 0<br>-1.5 -1 -0.5 0 0.5<br>Approximate distances	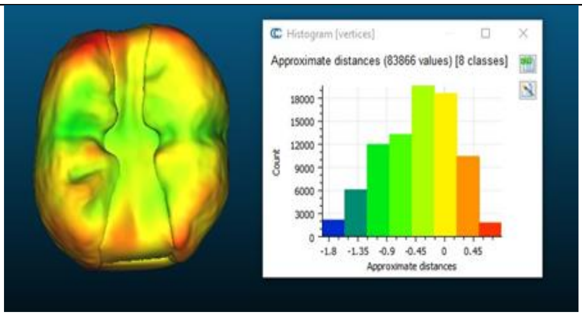Histogram [vertices]<br>Approximate distances (83866 values) [8 classes]<br>Count<br>18000 15000 12000 9000 6000 3000 0<br>-1.8 -1.35 -0.9 -0.45 0 0.45<br>Approximate distances
11	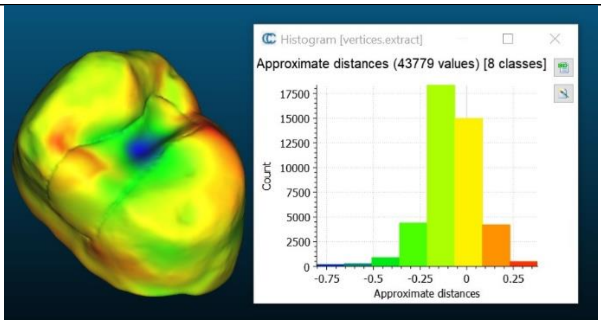Histogram [vertices.extract]<br>Approximate distances (43779 values) [8 classes]<br>Count<br>17500 15000 12500 10000 7500 5000 2500 0<br>-0.75 -0.5 -0.25 0 0.25<br>Approximate distances	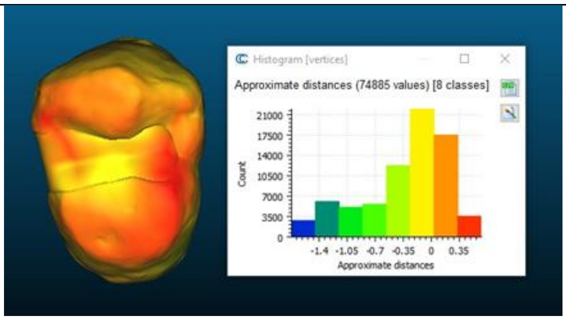Histogram [vertices]<br>Approximate distances (74885 values) [8 classes]<br>Count<br>21000 17500 14000 10500 7000 3500 0<br>-1.4 -1.05 -0.7 -0.35 0 0.35<br>Approximate distances
12	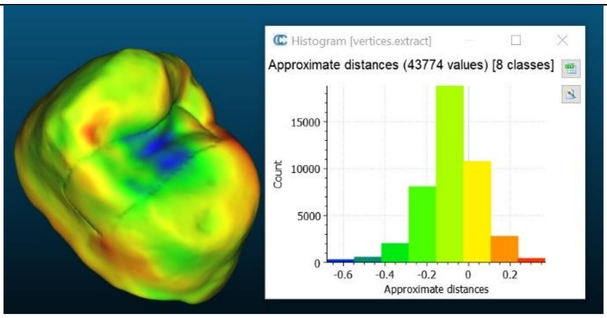Histogram [vertices.extract]<br>Approximate distances (43774 values) [8 classes]<br>Count<br>15000 10000 5000 0<br>-0.6 -0.4 -0.2 0 0.2<br>Approximate distances	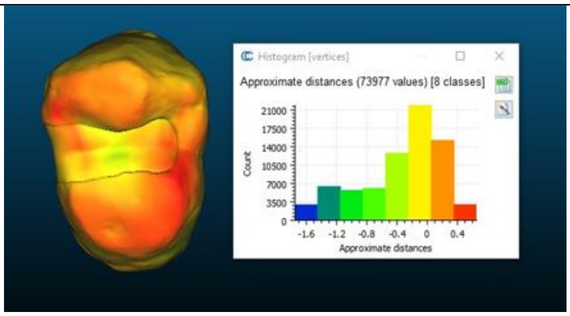Histogram [vertices]<br>Approximate distances (73977 values) [8 classes]<br>Count<br>21000 17500 14000 10500 7000 3500 0<br>-1.6 -1.2 -0.8 -0.4 0 0.4<br>Approximate distances

13	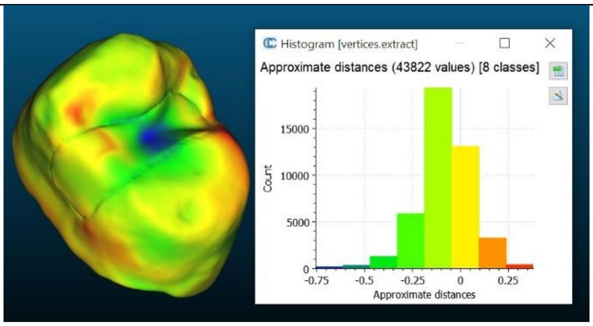Histogram [vertices.extract]<br>Approximate distances (43822 values) [8 classes]<br>Count<br>Approximate distances	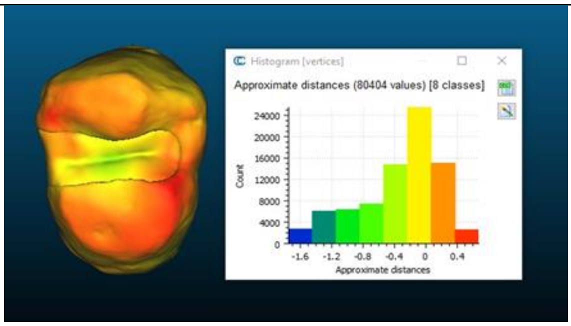Histogram [vertices]<br>Approximate distances (80404 values) [8 classes]<br>Count<br>Approximate distances
14	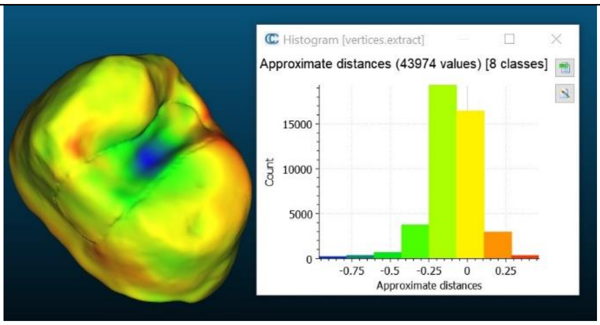Histogram [vertices.extract]<br>Approximate distances (43974 values) [8 classes]<br>Count<br>Approximate distances	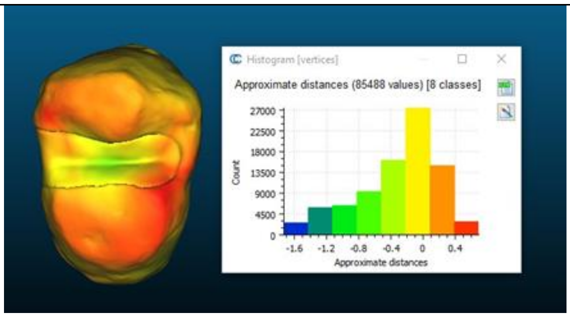Histogram [vertices]<br>Approximate distances (85488 values) [8 classes]<br>Count<br>Approximate distances
15	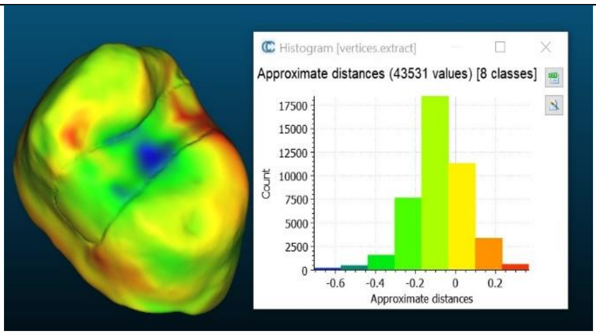Histogram [vertices.extract]<br>Approximate distances (43531 values) [8 classes]<br>Count<br>Approximate distances	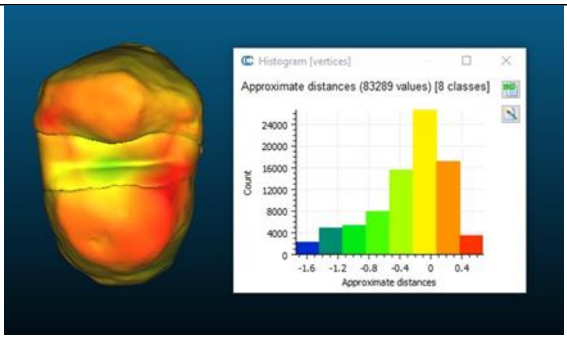Histogram [vertices]<br>Approximate distances (83289 values) [8 classes]<br>Count<br>Approximate distances

5. Dice Similarity Coefficient

Samples	Operator 1	Operator 2
1	0.939905	0.856353
2	0.938907	0.856627
3	0.939486	0.861130
4	0.938441	0.859198
5	0.940409	0.855673
6	0.851543	0.795881
7	0.848306	0.798396
8	0.851321	0.807343
9	0.848255	0.813377
10	0.847301	0.809659
11	0.945688	0.885706
12	0.944627	0.887758
13	0.945454	0.886824
14	0.941094	0.887798
15	0.943548	0.885934

e) Comparison made against virtually scanned and conventionally made Inlays

f) Hausdorff’s distance measured with onlays attached to the samples, to mitigate oversampling influenced inconsistencies

Phase 2: Virtual parameter evaluation outcomes for Onlay designs in Workflow 2

1. Mesh Surface Area

Samples	Operator 1	Operator 2
1	426.928253	430.436798
2	424.066437	431.604492
3	424.579041	427.127106
4	428.598816	432.335907
5	430.906494	435.725677
6	307.510345	294.779083
7	307.273529	303.402130
8	314.357147	313.271332
9	311.901794	305.985962
10	314.920776	307.528015
11	147.455688	138.771866
12	150.773102	143.236542
13	149.348022	144.460999
14	152.624237	138.794815
15	151.248550	141.874084

2. Virtual Volume

Samples	Operator 1	Operator 2
1	272.618225	272.177826
2	270.235870	266.380890
3	275.402100	266.220123
4	273.465942	270.175659
5	272.020813	272.511047
6	171.640717	159.475891
7	170.571091	170.700424
8	174.787582	174.061813
9	171.496994	169.184479
10	173.452301	165.916962
11	63.030849	56.233894
12	65.805733	61.184933
13	63.259949	59.142876
14	65.927345	56.701118
15	66.081116	58.773804

3. Hausdorff's Distance

Samples	Operator 1	Operator 2
1	0.030160 ±0.190322	-0.338327 ±0.716818
2	0.026761 ±0.192148	-0.430538 ±0.724948
3	0.030366 ±0.191116	-0.347961 ±0.726917
4	0.030383 ±0.189651	-0.338015 ±0.721700
5	0.025272 ±0.225857	-0.332609 ±0.723064
6	-0.083200 ±0.162333	-0.119360 ±0.188511
7	-0.082895 ±0.159217	-0.328682 ±0.588457
8	-0.066249 ±0.156153	-0.073351 ±0.157500
9	-0.075512 ±0.158511	-0.319018 ±0.575704
10	-0.065883 ±0.154818	-0.322096 ±0.573796
11	-0.072291 ±0.147974	-0.200725 ±0.502298
12	-0.056789 ±0.129332	-0.206426 ±0.499225
13	-0.062576 ±0.130747	-0.217944 ±0.498508
14	-0.058713 ±0.140052	-0.221223 ±0.498606
15	-0.057320 ±0.129853	-0.229074 ±0.505443

g) Comparison made against virtually scanned and conventionally made Onlays

h) Hausdorff's distance measured with onlays attached to the samples, to mitigate oversampling influenced inconsistencies

4. Color map of Hausdorff's distance

Specimen	Operator 1	Operator 2
1	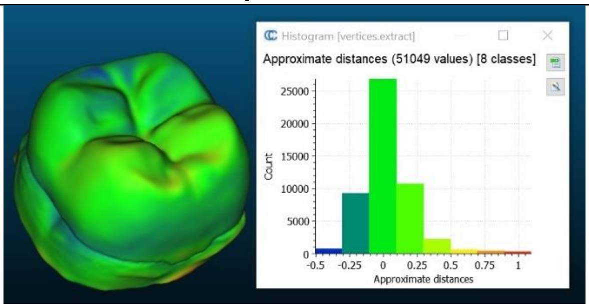	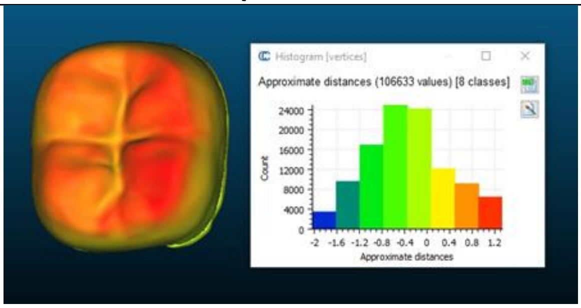
2	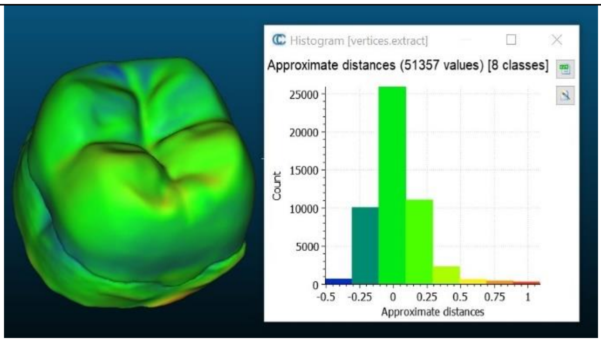	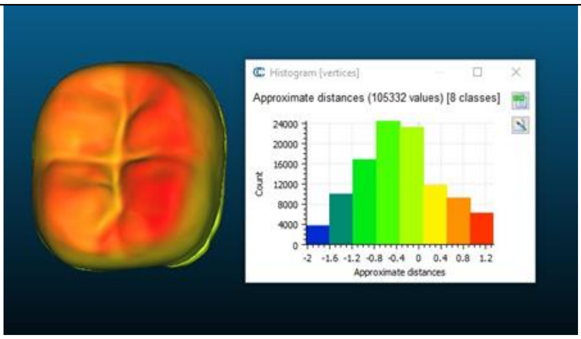
3	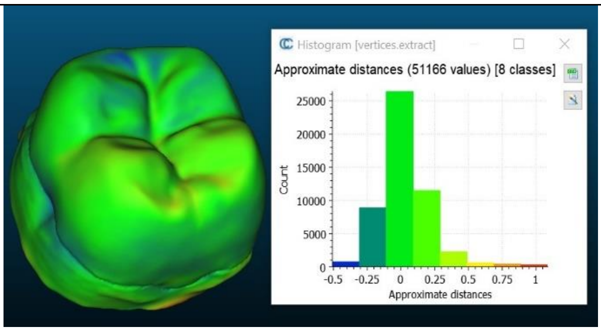	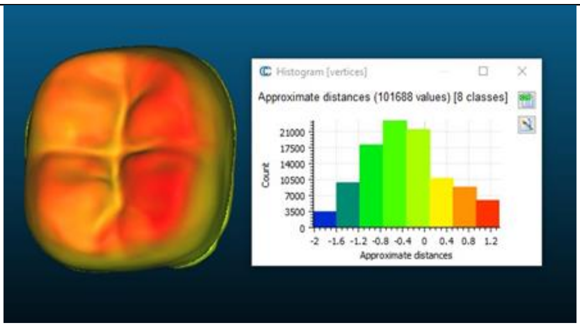
4	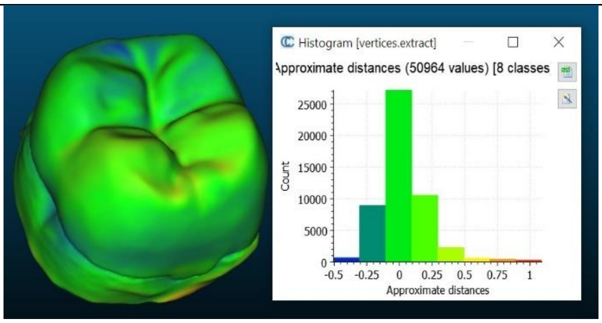	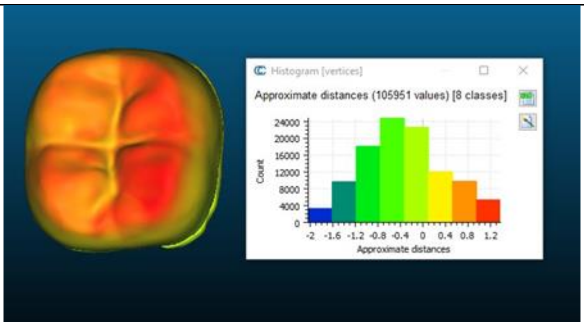

<table>
<tr>
<td>5</td>
<td>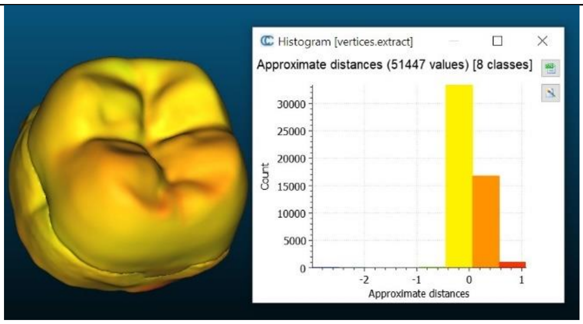
</td>
<td>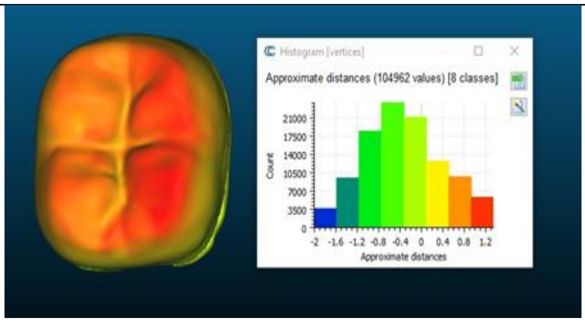
</td>
</tr>
<tr>
<td>6</td>
<td>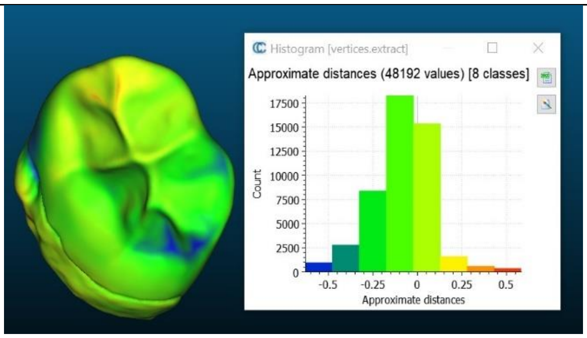
</td>
<td>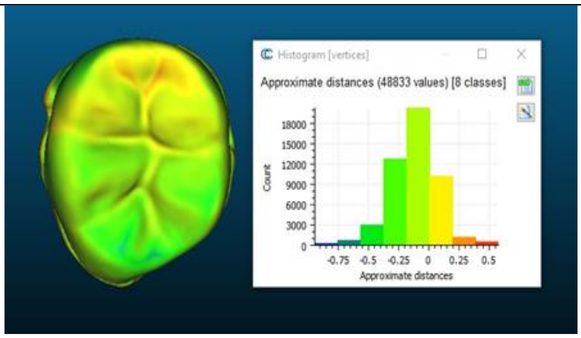
</td>
</tr>
<tr>
<td>7</td>
<td>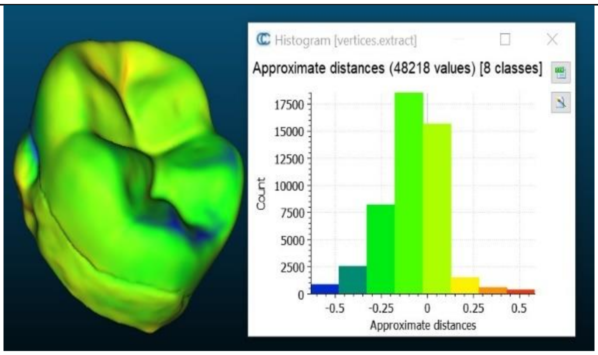
</td>
<td>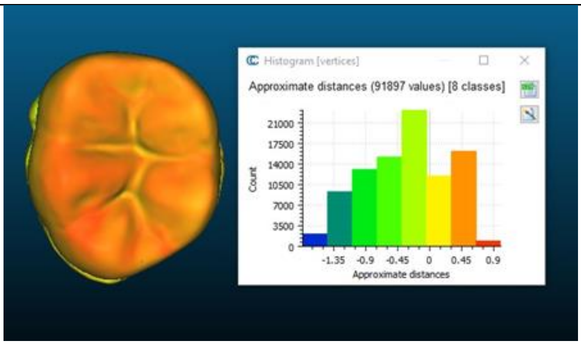
</td>
</tr>
<tr>
<td>8</td>
<td>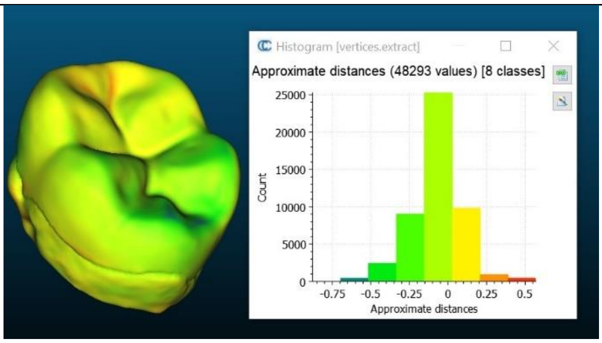
</td>
<td>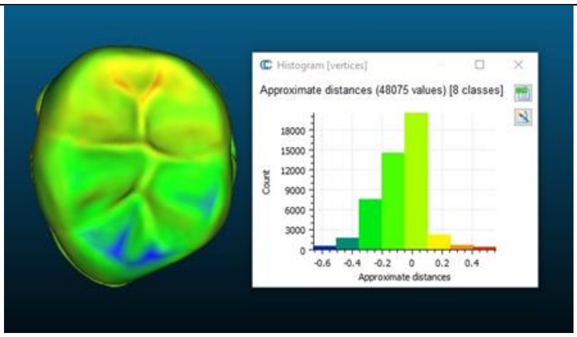
</td>
</tr>
</table>

9	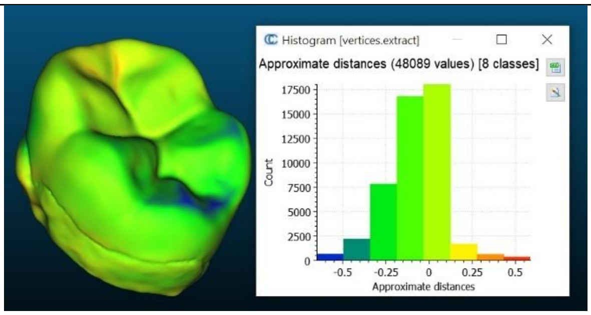Histogram [vertices.extract]<br>Approximate distances (48089 values) [8 classes]<br>Count<br>Approximate distances	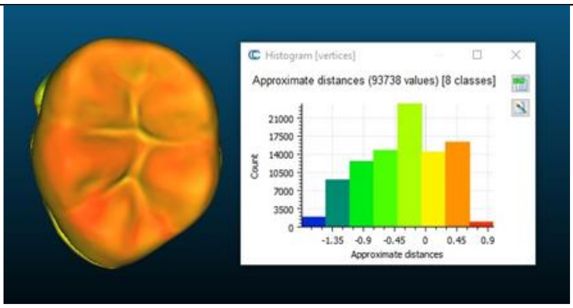Histogram [vertices]<br>Approximate distances (93738 values) [8 classes]<br>Count<br>Approximate distances
10	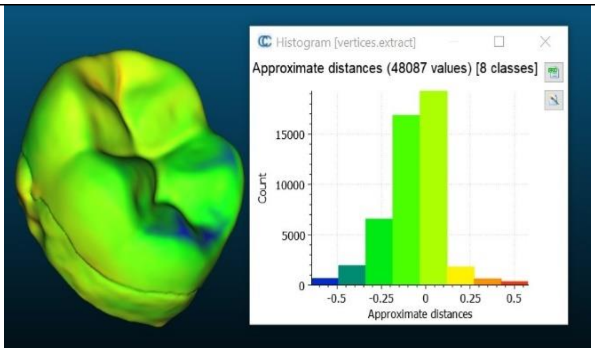Histogram [vertices.extract]<br>Approximate distances (48087 values) [8 classes]<br>Count<br>Approximate distances	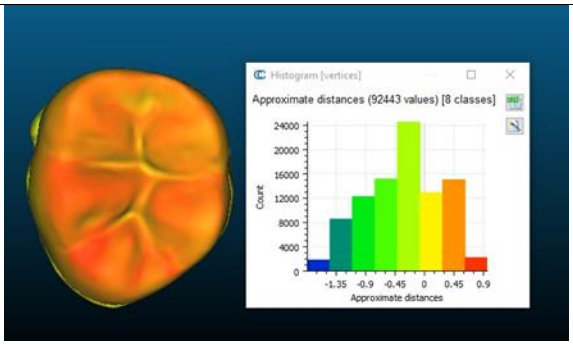Histogram [vertices]<br>Approximate distances (92443 values) [8 classes]<br>Count<br>Approximate distances
11	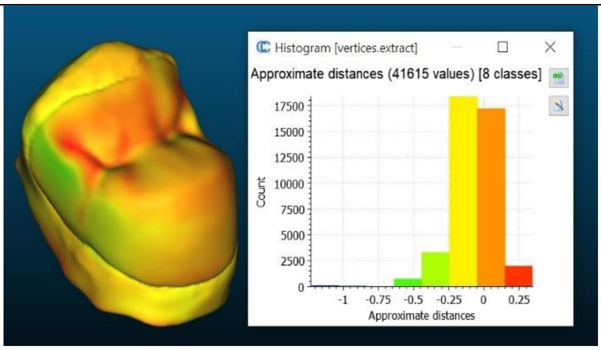Histogram [vertices.extract]<br>Approximate distances (41615 values) [8 classes]<br>Count<br>Approximate distances	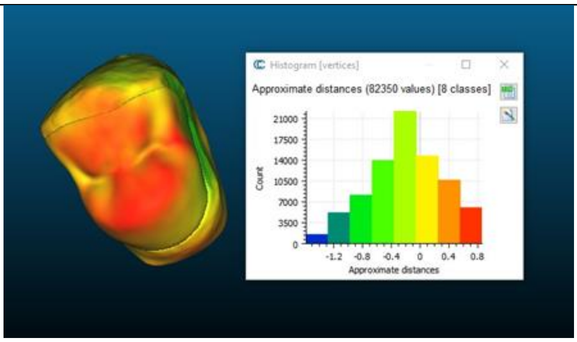Histogram [vertices]<br>Approximate distances (82350 values) [8 classes]<br>Count<br>Approximate distances
12	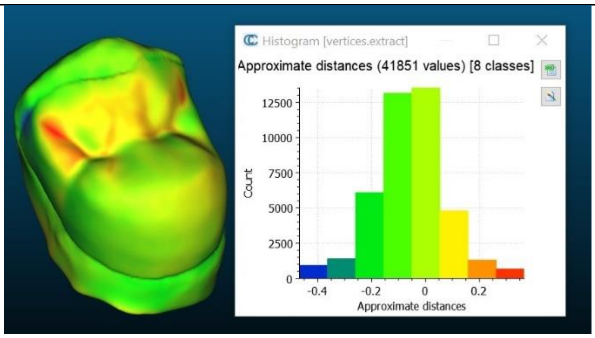Histogram [vertices.extract]<br>Approximate distances (41851 values) [8 classes]<br>Count<br>Approximate distances	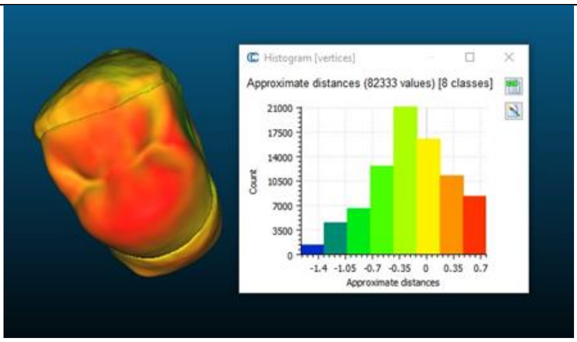Histogram [vertices]<br>Approximate distances (82333 values) [8 classes]<br>Count<br>Approximate distances

<table>
<tr><td>13</td><td>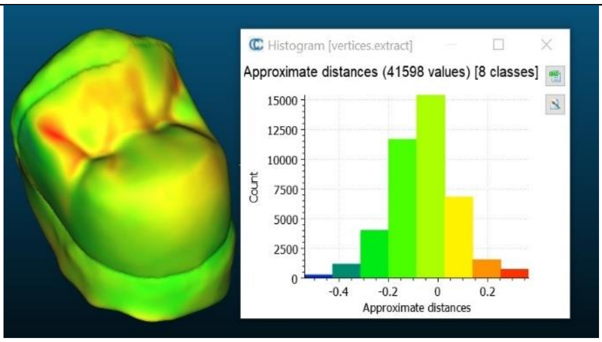
</td><td>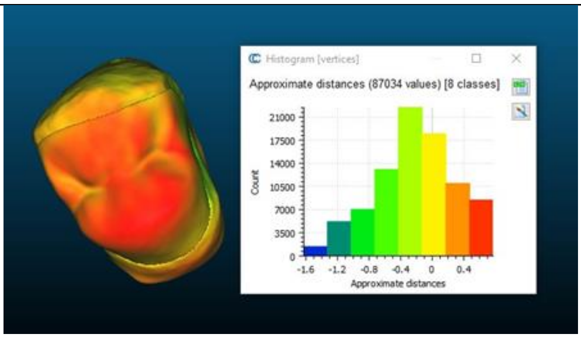
</td></tr>
<tr><td>14</td><td>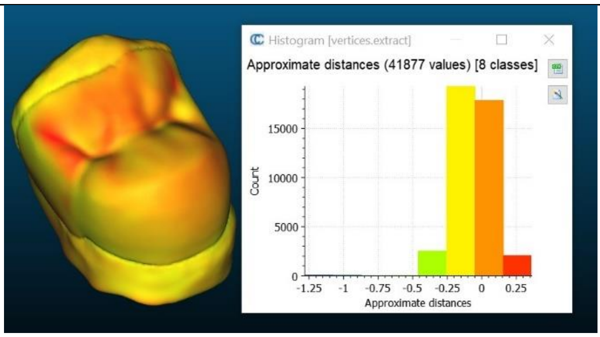
</td><td>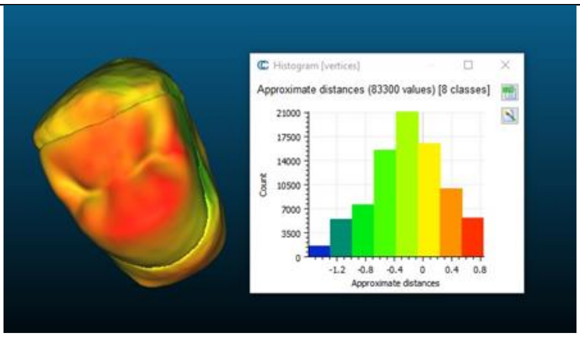
</td></tr>
<tr><td>15</td><td>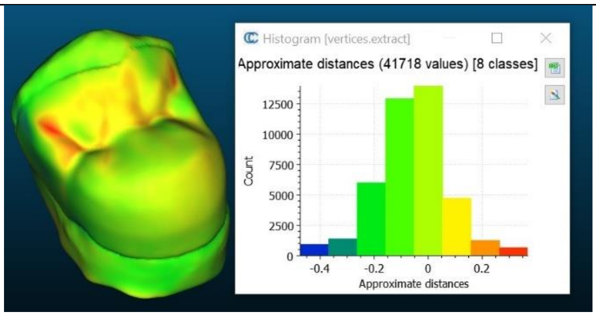
</td><td>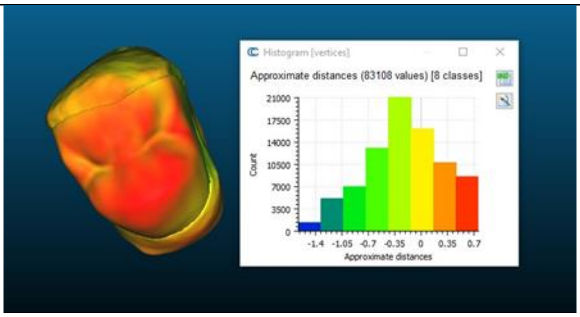
</td></tr>
</table>

5. Dice Similarity Coefficient

Samples	Operator 1	Operator 2
1	0.957661	0.829373
2	0.956816	0.816703
3	0.943675	0.822031
4	0.958078	0.817534
5	0.957369	0.814989
6	0.949892	0.938788
7	0.950585	0.840246
8	0.955312	0.952830
9	0.952704	0.844812
10	0.955262	0.842167
11	0.949337	0.844596
12	0.953480	0.852279
13	0.951866	0.855818
14	0.952832	0.847375
15	0.953343	0.850619

c) Comparison made against virtually scanned and conventionally made Onlays

d) Hausdorff's distance measured with onlays attached to the samples, to mitigate oversampling influenced inconsistencies

Phase 3: 3-dimensional convolutional neural network sensitivity and specificity

Tooth Preparation Model					
**Validation File No.**	**Predicted Label**	**Actual result**	**False Positive**	**True Positive**	**False Negatives**
	Onlay	Inlay	1	0	0
	Onlay	Inlay	1	0	0
	Onlay	Inlay	1	0	0
	Onlay	Inlay	1	0	0
	Onlay	Inlay	1	0	0
	Onlay	Onlay	0	1	0
	Onlay	Onlay	0	1	0
	Onlay	Onlay	0	1	0
	Onlay	Onlay	0	1	0
	Onlay	Onlay	0	1	0
Prostheses Model					
**Validation File No.**	**Predicted Label**	**Actual result**	**False Positive**	**True Positive**	**False Negatives**
	Onlay	Inlay	1	0	0
	Inlay	Inlay	0	0	0
	Inlay	Inlay	0	0	0
	Inlay	Inlay	0	0	0
	Inlay	Inlay	0	0	0
	Onlay	Onlay	0	1	0
	Onlay	Onlay	0	1	0
	Onlay	Onlay	0	1	0
	Onlay	Onlay	0	1	0
	Onlay	Onlay	0	1	0

Supplementary Figures

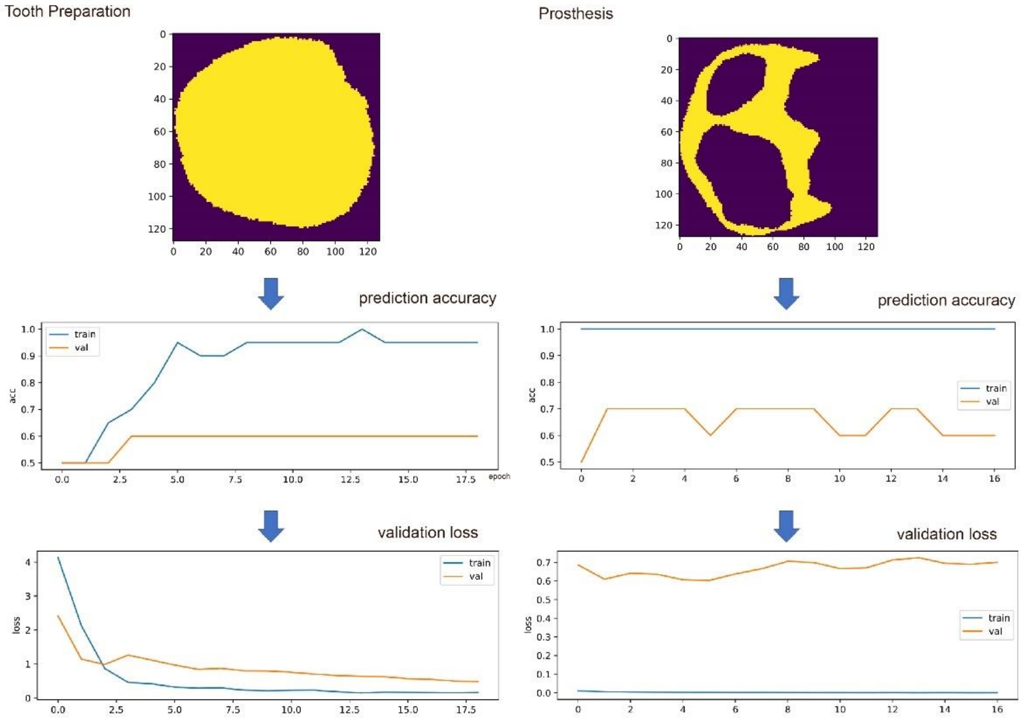

Supplementary (S) Figure 1: Graphical outcomes of prediction accuracy and validation loss for 3D models of tooth preparation (left) and corresponding prostheses (right)

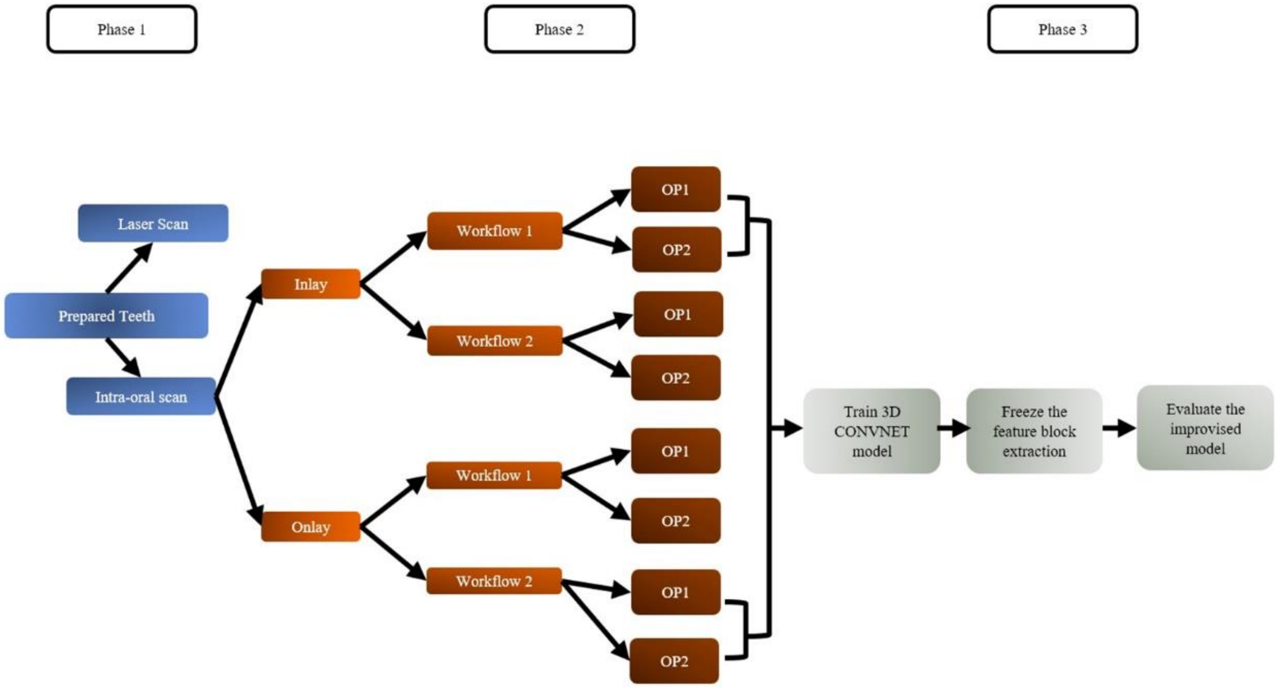

Supplementary (S) Figure 2: Flowchart summary of the study design. (OP1 = Operator 1, OP2 = Operator 2)

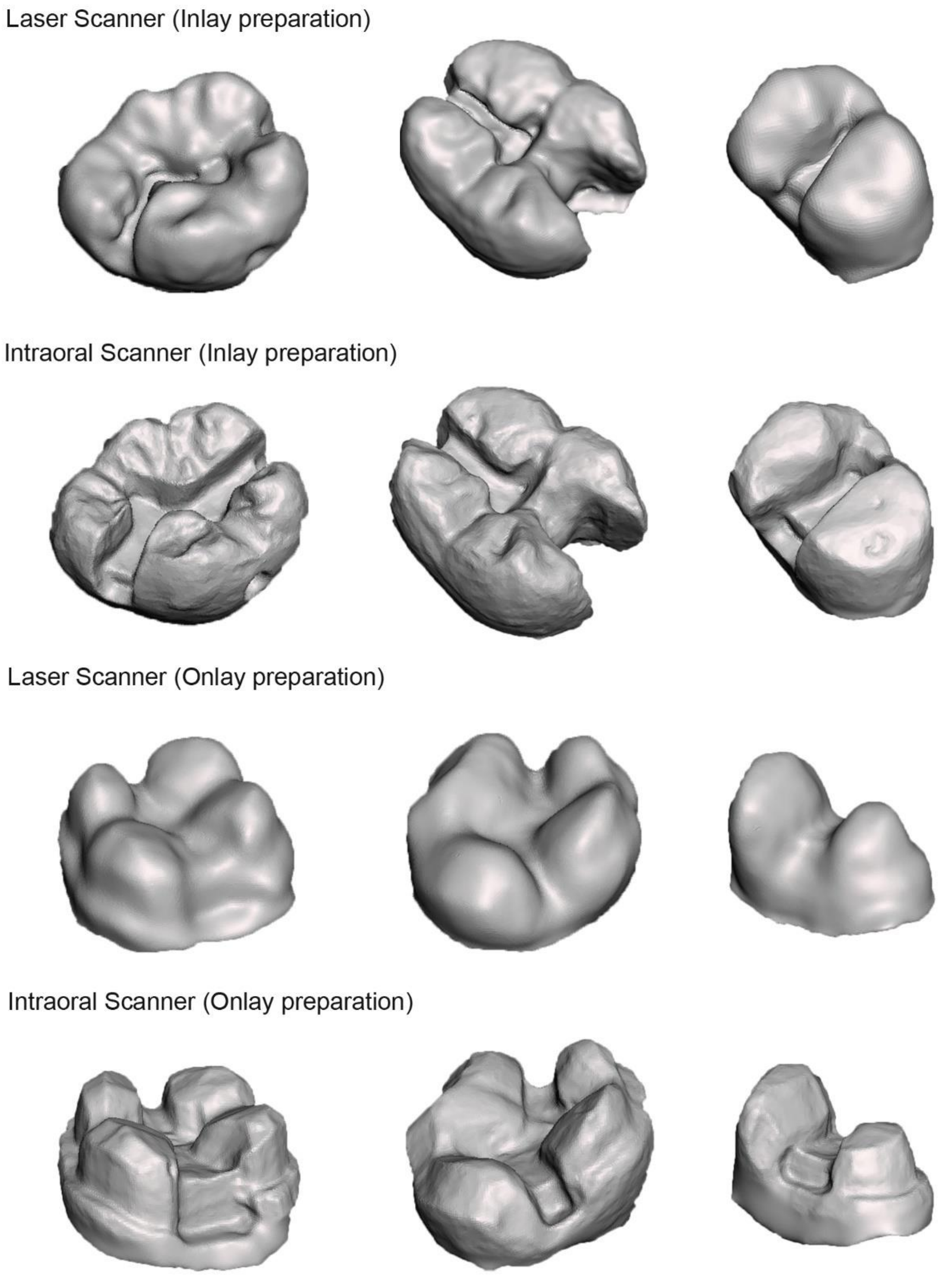

Supplementary (S) Figure 3: Comparison of 3D scan quality of tooth preparations for partial dental crowns.

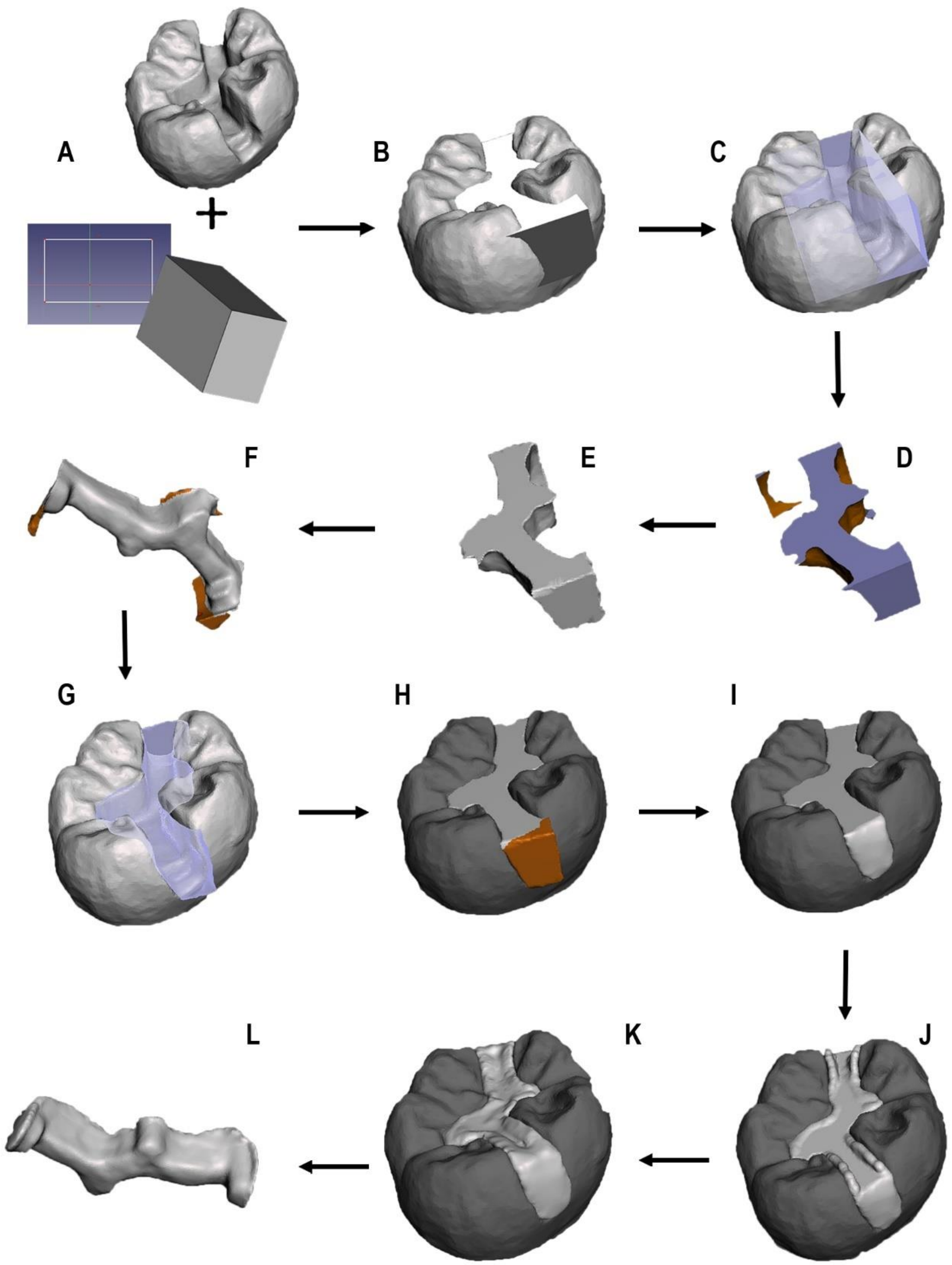

Supplementary (S) Figure 4: Workflow 2 for inlay design: A) create 3D block in CAD, B) load block into tooth preparation, C) ensure block occupies entire defect, D) perform Boolean difference, E) perform ‘make solid’ function, F) discard excess rough edge, G) ensure proper 3D seal and contact, H) reduce peripheral excess, I) sculpt tooth contour, J) inflate edges to create marginal ridge and cuspal contact, K) further draw, shrink and smooth inflation to contour the occlusal surface, L) finalize the design.

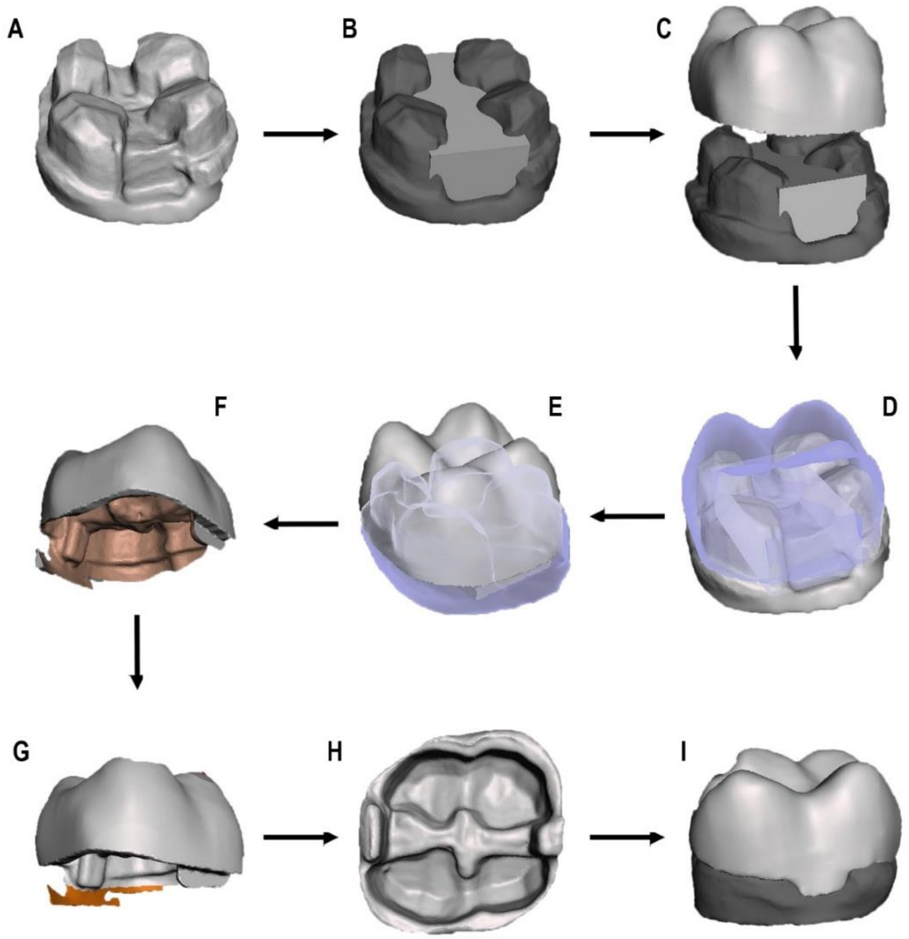

Supplementary (S) Figure 5: Workflow 2 for onlay design: A) import onlay preparation, B) superimpose block, C) place crown template over block and preparation model, D) combine block and crown template, E) perform 'make solid' on crown and block, F) perform Boolean difference between combined structure and tooth preparation, G) remove excess and autofix holes, H) sculpt, trim and smoothen the contour, I) finalize the design and check fit.

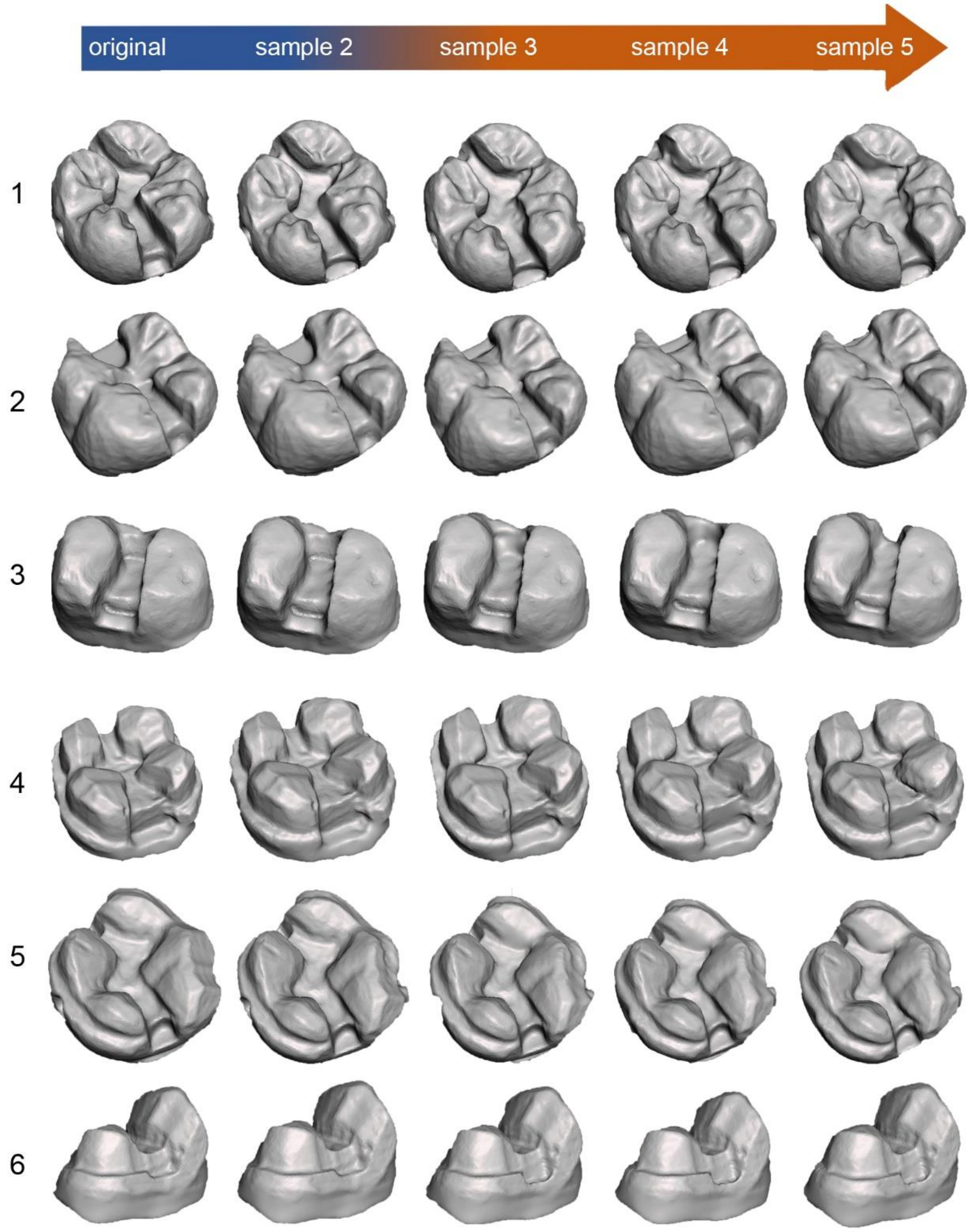

Supplementary (S) Figure 6: oversampling of existing 3D models of tooth preparation.

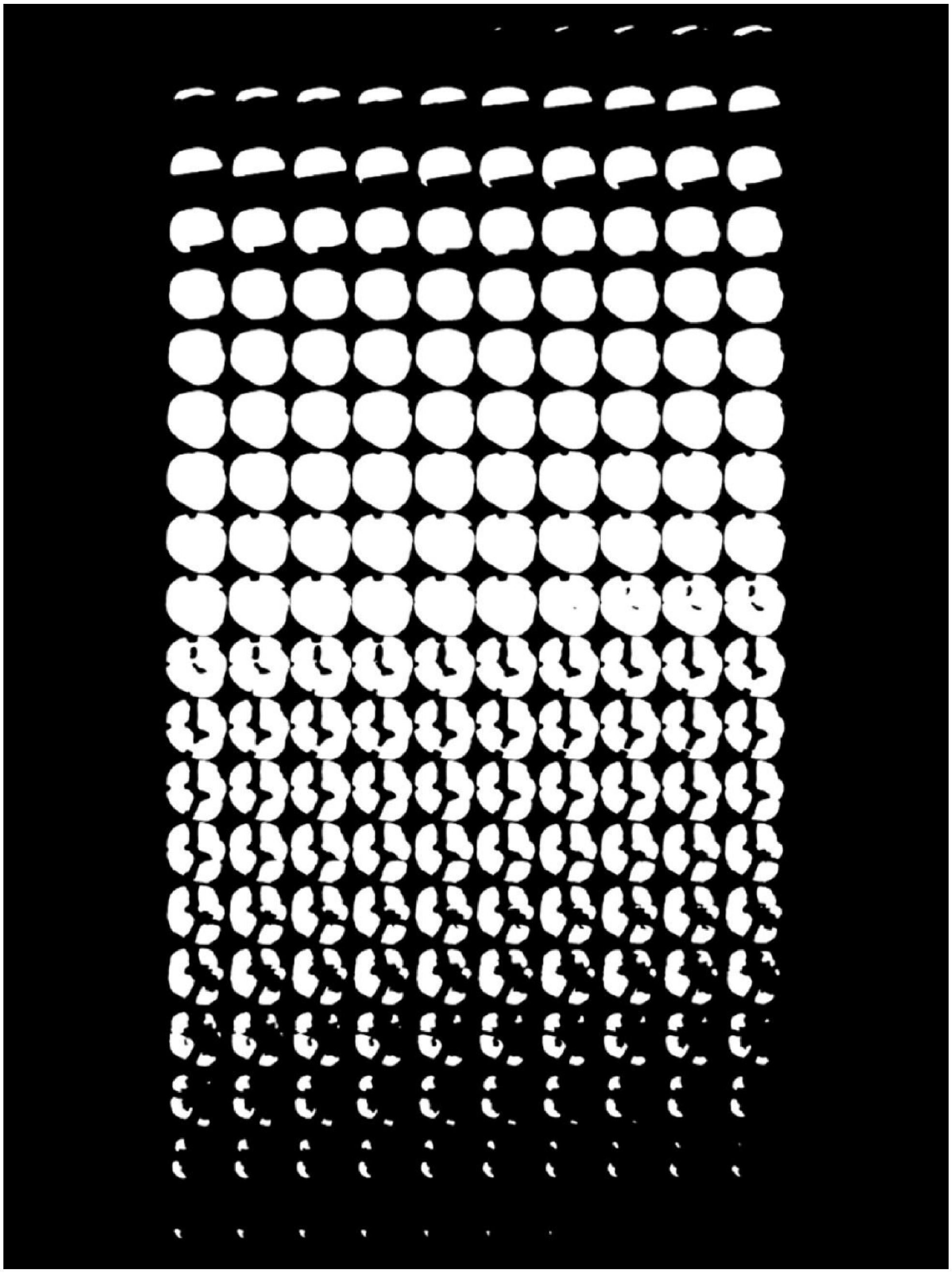

Supplementary (S) Figure 7: Stacked view of one 3D model of tooth preparation sliced into 2D segments.

Supplementary Tables

Supplementary Table 1: Analysis of variance (n=116) for virtual volume

List of independent factors:

1. **Type of Prosthesis**: F(df)= 112.91 (1), *P*= <.001

   Inlay = Mean ±SD =36.85 ±11.93
   Onlay = Mean ±SD =165.38 ±87.41

2. **Virtual Workflow**: F(df) = 0.001 (1), *P*= .973

   Workflow 1 (3matics) = Mean ±SD =103.06 ±89.75
   Workflow 2 (Meshmixer) = Mean ±SD =103.60 ±91.58

3. **Clinical Operator**: F(df) <.001 (1), *P*= .984

   Operator 1 = Mean ±SD =103.21 ±90.90
   Operator 2 = Mean ±SD =103.45 ±90.43

**Inlay**		
	**Operator 1** *(Mean ±SD)*	**Operator 2** *(Mean ±SD)*
**Workflow 1**	38.20 ±12.78	39.16 ±12.75
**Workflow 2**	35.47 ±11.41	34.56 ±11.41
**Onlay**		
	**Operator 1** *(Mean ±SD)*	**Operator 2** *(Mean ±SD)*
**Workflow 1**	160.32 ±92.52	165.96 ±88.84
**Workflow 2**	169.99 ±87.90	165.26 ±89.29

Interaction effect

1. Type of Prosthesis vs. Virtual Workflow: F(df) = 0.113 (1), *P*= .737
2. Type of Prosthesis vs. Clinical Operator: F(df) <.001 (1), *P*= .986
3. Virtual Workflow vs. Clinical Operator: F(df) = 0.064 (1), *P*= .801
4. Type of Prosthesis vs Virtual Workflow vs Clinical Operator: F(df) = 0.031(1), *P*= .861

Supplementary Table 2: Analysis of variance (n=116) for Hausdorff's Distance (in mm)

List of independent factors:

1. **Type of Prosthesis**: F(df)= 1.81 (1), *P*= .181

   Inlay = Mean ±SD = 0.23 ±0.11
   Onlay = Mean ±SD = 0.21 ±0.12

2. **Virtual Workflow**: F(df) = 15.62 (1), *P* <.001

   Workflow 1 (3matics) = Mean ±SD = 0.25 ±0.08
   Workflow 2 (Meshmixer) = Mean ±SD = 0.19 ±0.14

3. **Clinical Operator**: F(df) = 37.27 (1), *P* <.001

   Operator 1 = Mean ±SD = 0.17 ±0.12
   Operator 2 = Mean ±SD = 0.27 ±0.09

	**Inlay**	
	**Operator 1** *(Mean ±SD)*	**Operator 2** *(Mean ±SD)*
**Workflow 1**	0.24 ±0.09	0.23 ±0.08
**Workflow 2**	0.15 ±0.12	0.30 ±0.09
	**Onlay**	
	**Operator 1** *(Mean ±SD)*	**Operator 2** *(Mean ±SD)*
**Workflow 1**	0.26 ±0.07	0.27 ±0.07
**Workflow 2**	0.04 ±0.05	0.27 ±0.10

Interaction effect

1. Type of Prosthesis vs. Virtual Workflow: F(df) = 10.38 (1), *P*= .002
2. Type of Prosthesis vs. Clinical Operator: F(df) = 2.30 (1), *P*= .132
3. Virtual Workflow vs. Clinical Operator: F(df) = 34.90 (1), *P* <.001
4. Type of Prosthesis vs Virtual Workflow vs Clinical Operator: F(df) = 0.995(1), *P*= .321

Supplementary Table 3: Analysis of variance (n=116) for Dice Similarity Coefficient

List of independent factors:

1. **Type of Prosthesis**: F(df)= 1.34 (1), *P*= .250

   Inlay = Mean ±SD = 0.87 ±0.04
   Onlay = Mean ±SD = 0.88 ±0.06

2. **Virtual Workflow**: F(df) = 20.95 (1), *P* <.001

   Workflow 1 (3matics) = Mean ±SD = 0.86 ±0.04
   Workflow 2 (Meshmixer) = Mean ±SD = 0.89 ±0.06

3. **Clinical Operator**: F(df) = 45.95 (1), *P* <.001

   Operator 1 = Mean ±SD = 0.90 ±0.05
   Operator 2 = Mean ±SD = 0.85 ±0.03

**Inlay**		
	**Operator 1** *(Mean ±SD)*	**Operator 2** *(Mean ±SD)*
**Workflow 1**	0.86 ±0.04	0.87 ±0.03
**Workflow 2**	0.91 ±0.05	0.85 ±0.04
**Onlay**		
	**Operator 1** *(Mean ±SD)*	**Operator 2** *(Mean ±SD)*
**Workflow 1**	0.87 ±0.05	0.84 ±0.03
**Workflow 2**	0.95 ±0.01	0.85 ±0.04

Interaction effect

1. Type of Prosthesis vs. Virtual Workflow: F(df) = 6.21 (1), *P*= .014
2. Type of Prosthesis vs. Clinical Operator: F(df) = 5.81 (1), *P*= .018
3. Virtual Workflow vs. Clinical Operator: F(df) = 29.78 (1), *P* <.001
4. Type of Prosthesis vs Virtual Workflow vs Clinical Operator: F(df) = .393 (1), *P*= .532
